# Causal effects of hypertensive disorders of pregnancy on future gynecologic tumors: A two‐sample Mendelian randomization study

**DOI:** 10.1002/cam4.7300

**Published:** 2024-05-27

**Authors:** Le Zhou, Xinghui Liu, Guolin He, Chuntang Sun

**Affiliations:** ^1^ Department of Gynecology and Obstetrics West China Second University Hospital, Sichuan University Chengdu China; ^2^ Key Laboratory of Obstetrics & Gynecologic and Pediatric Diseases and Birth Defects of Ministry of Education West China Second University Hospital, Sichuan University Chengdu China

**Keywords:** breast cancer, endometrial cancer, hypertensive disorders in pregnancy, Mendelian randomization, preeclampsia

## Abstract

**Background:**

Numerous observational studies have investigated the potential link between hypertensive disorders of pregnancy (HDPs) and the subsequent risks of gynecologic tumors, yet the findings have been inconsistent. In this study, we utilized Mendelian randomization (MR) approach to assess the influence of HDPs on the future risks of ovarian, cervical, endometrial, and breast cancer and uterine fibroids, controlling for confounding factors.

**Methods:**

The genome‐wide association studies (GWAS) summary data relevant to HDPs was obtained from the FinnGen databases (10,736 cases and 136,325 controls). Gynecologic tumor outcomes were extracted from the IEU Open GWAS project and UK Biobank (47,690 cases and 1, 092,073 controls). The inverse variance weighted (IVW) approach was selected as the principal method for MR analysis, supplemented by MR‐Egger, weighted median, weighted model, simple model methods, MR pleiotropy residual sum and outlier (MR‐PRESSO) test, and leave‐one‐out method. Multivariate MR (MVMR) analysis was conducted after adjusting systolic blood pressure (SBP), body mass index (BMI) and type 2 diabetes mellitus (T2DM).

**Results:**

Our univariate MR analysis (UVMR) results revealed no significant relationship between HDPs and the risks of ovarian cancer (odds ratio [OR] = 0.924, *p* = 0.360), cervical cancer (OR = 1.230, *p* = 0.738), endometrial cancer (OR = 1.006, *p* = 0.949), uterine fibroids (OR = 1.155, *p* = 0.158), and breast cancer (OR = 0.792, *p* = 0.241) by IVW test. Similar results were observed in gestational hypertension and preeclampsia/eclampsia. Additionally, our study detected neither heterogeneity nor pleiotropy. MVMR analysis also provided no evidence of a causal association between HDPs and common gynecologic tumors after adjusting SBP, BMI, and T2DM.

**Conclusion:**

We discovered no causal relationship between HDPs and ovarian, cervical, endometrial, breast cancer, and uterine fibroids in European populations. However, present analysis did not explore the effect of HDPs on the subtypes of gynecologic tumors across varied ethnic populations, which may require additional research.

## 1 INTRODUCTION

Hypertensive disorders in pregnancy (HDPs), characterized by the onset of elevated blood pressure during gestation, affect an estimated 4.1%–19.4% of pregnancies worldwide.^1^ As the second biggest cause of maternal death, HDPs also pose a substantial risk to newborn health.^2^ HDPs can be categorized into three specific diagnoses: preeclampsia or eclampsia, gestational hypertension, and any of these conditions occurring concurrently with chronic hypertension.^3^ In addition to the direct impact on maternal and fetal health, HDPs are linked to considerable alterations in future disease risk for the mother. Numerous systematic reviews and meta‐analyses have conclusively demonstrated an elevated future cardiovascular disease risk among women who have experienced HDPs.^4^, ^5^

Insufficient knowledge exists about the correlation between HDPs and the risk of cancer. Most prior research has largely focused on exploring the association between preeclampsia and breast cancer, often yielding inconsistent results.^6^, ^7^ A recent meta‐analysis failed to establish a significant link between maternal breast cancer susceptibility and either preeclampsia or gestational hypertension.^8^ However, findings from later studies have been inconsistent. A comprehensive cohort investigation reported a diminished risk of breast cancer in women with a history of HDPs.^9^ Several other researches have echoed this finding, suggesting a risk reduction in the range of 10%–20%.^6^, ^7^, ^10^ Nevertheless, the potential relationship between HDPs and other gynecologic tumors, such as ovarian, cervical, and endometrial cancer, as well as uterine fibroids, is poorly understood. Current research on these gynecologic tumors has yielded inconsistent outcomes.^11^, ^12^ Evidence from the European Prospective Investigation into Cancer (EPIC)‐Norfolk cohort suggested no association between HDPs and future risk of ovarian, endometrial, and breast cancers.^11^ Another nationwide cohort study revealed that a history of HDPs was significantly related to decreased incidence of breast cancer and with increased incidence of endometrial cancer.^12^ Furthermore, the criteria for identifying HDPs varied across research. Powell et al. considered individuals with reports of gestational hypertension and/or preeclampsia as having HDPs.^9^ By contrast, Goldberg et al. defined women with any self‐reported preeclampsia, eclampsia, or high blood pressure during pregnancy as those with a history of HDPs.^13^ Unquestionably, determining the causal association between HDPs and future gynecologic tumors may have far‐reaching influence on cancer prevention strategies. Given the potential for residual confounders, the infeasibility of randomized controlled trials (RCTs), and the scarcity of large cohort studies, innovative methodologies are urgently required to determine causality.

Mendelian randomization (MR) serves as a powerful method for establishing causal inference by utilizing single nucleotide polymorphisms (SNPs) as instrumental variables (IVs).^14^ Analogous to treatment randomization in clinical trials, genetic variants are assigned randomly during gamete formation and conception, uninfluenced by external factors.^15^ This results in efficacious randomization of high or low genetic risks for diseases, thus minimizing the possibility of confusion and reverse causality, akin to RCTs. Summary data from genome‐wide association studies (GWAS) are more readily available and typically extensive for two‐sample MR analysis, which serves to strengthen genetic interpretation of IVs on exposure and enhances the precision and dependability of analysis outcomes.^16^ Consequently, this study aims to perform a two‐sample MR analysis utilizing pooled data from GWAS to explore the relationship between HDPs and the risks of ovarian, cervical, endometrial, and breast cancer, as well as uterine fibroids.

## 2 METHODS

### 2.1 Study design and data sources

**FIGURE 1:**
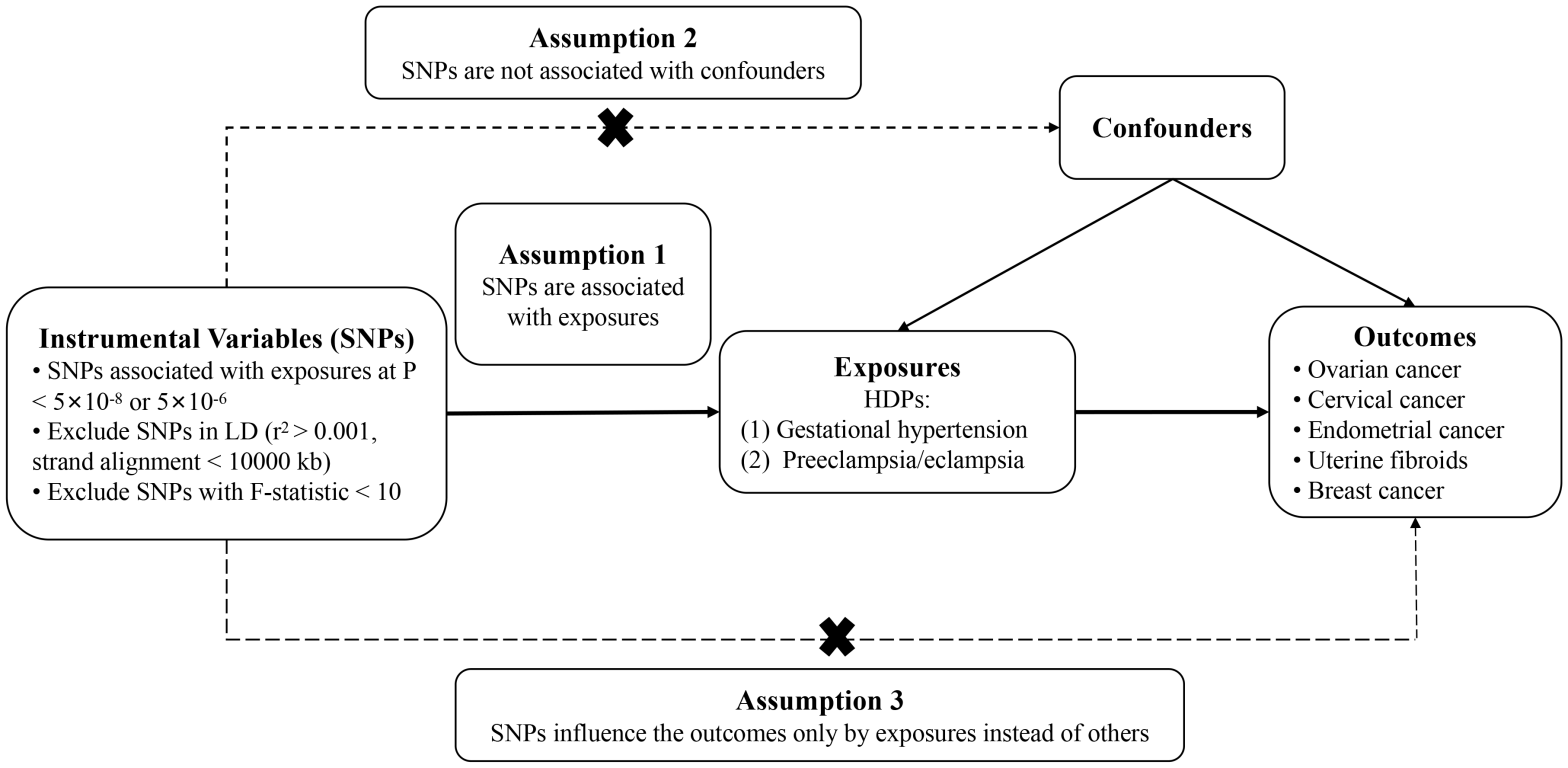
Study design diagram and three assumptions of Mendelian randomization (MR).

The present study used a two‐sample MR analysis to ascertain the causal relationship between exposures and outcomes. For the causal predictions produced from the two‐sample MR analysis to hold validity, the SNPs utilized in MR must meet three essential prerequisites: (1) the SNPs were associated with HDPs, gestational hypertension, or preeclampsia/eclampsia; (2) the SNPs were not linked to confounders; (3) the SNPs impacted the outcomes solely via the exposure factors. The MR study design was shown in Figure 1. The present research was based on publicly accessible summary data from large GWAS, which were from European descent. The ethical approval of all included GWAS data has been accomplished and approved by the Ethics Committee. As the present study only extracted data from GWAS databases for analysis, an ethical review was not necessitated.

The study encompassed three exposure variables: HDPs in aggregate and its two subcategories, preeclampsia/eclampsia and gestational hypertension. The estimates for genetic connections were derived from the sixth data dissemination of GWAS summary data by the FinnGen consortium, which was made available on January 24,2022. The dataset contained HDPs (10,736 cases and 136,325 controls), gestational hypertension (5240 cases and 136,325 controls), and preeclampsia/eclampsia (4743 cases and 136,325 controls). The average age at the initial event ranged from 29.06 to 30.08 years.^17^

By consulting the PhenoScanner database (http://www.phenoscanner.medschl.cam.ac.uk/), we considered systolic blood pressure (SBP), body mass index (BMI), and type 2 diabetes mellitus (T2DM) as potential confounders based on their known or plausible effects on HDPs.^17^, ^18^ The summary statistics for SBP (422,713 European ancestry individuals),^19^ BMI (407, 609 British ancestry individuals),^20^ and T2DM (468,298 European ancestry individuals)^21^ were obtained from the publications based on the UK Biobank.

The GWAS summary data relevant to ovarian cancer, which is part of the Ovarian Cancer Association Consortium (OCAC), was procured from the IEU Open GWAS project (https://gwas.mrcieu.ac.uk/), encompassing 25,509 cases and 40,941 controls. The summary data on cervical cancer (258 cases and 247,282 controls), uterine fibroids (7187 cases and 240,353 controls), and breast cancer (1830 cases and 454,518 controls) were obtained from results of the GWAS in the UK Biobank.^22^ SNPs associated with endometrial cancer were sourced from the endometrial GWAS data, which collectively included 12,906 cases and 108,979 controls (Table 1).^23^

**TABLE 1:** Summary of the genome‐wide association studies (GWAS) included in this MR study.

Trait	Dataset	Sample size	Number of SNPs	Population	Consortium	Sex	Year
Exposures							
Hypertensive disorders of pregnancy	finngen_R6_O15_OEDEM_PROTUR_HYPERT	10,736 cases and 136,325 controls	16,354, 723	European	FinnGen	Females	2022
Gestational hypertension	finngen_R6_O15_GESTAT_HYPERT	5240 cases and 136,325 controls	16,354, 645	European	FinnGen	Females	2022
Pre‐eclampsia or eclampsia	finngen_R6_O15_PRE_OR_ECLAMPSIA	4743 cases and 136,325 controls	16,354, 667	European	FinnGen	Females	2022
Adjustments							
Systolic blood pressure	PMID: 34226706	422,713 European ancestry individuals	4,228, 468	European	UK Biobank	Males and females	2021
Body mass index	PMID: 34017140	407,609 British ancestry individuals	10,783, 680	European	UK Biobank	Males and females	2021
Type 2 diabetes mellitus	PMID: 29892013	468,298 European ancestry individuals	11,973, 400	European	UK Biobank	Males and females	2018
Outcomes							
Ovarian cancer	ieu‐a‐1120	25,509 cases and 40,941 controls	Approximately 530 k	European	OCAC	Females	2017
Cervical cancer	PMID: 34737426	258 cases and 247,282 controls	11,831, 390	European	UK Biobank	Females	2021
Endometrial cancer	PMID: 30093612	12,906 cases and 108,979 controls	9,525, 227	European	ECAC, E2C2	Females	2018
Uterine fibroids	PMID: 34737426	7187 cases and 240,353 controls	11, 831, 390	European	UK Biobank	Females	2021
Breast cancer	PMID: 34737426	1830 cases and 454,518 controls	11,831, 294	European	UK Biobank	Females	2021

Abbreviation: HDPs, hypertensive disorders of pregnancy.

### 2.2 Instrumental variable selection

IVs were selected on the basis of their correlation with each exposure at a threshold of *p* < 5 × 10^−8^. We implemented linkage disequilibrium (LD) using European ancestry reference data (1000 Genomes Project, *r* ^2^ = 0.001, strand alignment = 10,000 kb). For preeclampsia/eclampsia, the absence of SNPs at this threshold led us to escalate the *p* threshold tenfold until a variant was attainable, leading to a threshold of *p* < 5 × 10^−6^.^17^ Pleiotropy identification for each selected SNP was performed using Phenoscanner (http://www.phenoscanner.medschl.cam.ac.uk/). We included pleiotropic SNPs, but subsequently excluded them if horizontal pleiotropy was detected in sensitivity analysis. To evaluate the potency of the chosen IVs in explaining exposure traits, we computed *F*‐statistic. IVs with an *F*‐statistic less than 10 were excluded.^24^ Additionally, we harmonized the exposures and outcomes to exclude palindromic SNPs and confirm the uniformity of the effect alleles. Palindromic SNPs were defined as those possessing ambiguous minor allele frequencies falling between >0.45 and <0.55 and were subsequently removed from our dataset.^25^, ^26^ Details of the included SNPs were presented in Tables S1–S3.

### 2.3 MR analyses

Univariate MR analysis (UVMR) utilized genetic variants as IVs to assess the causal impact of HDPs on common gynecologic tumors. We selected the inverse variance weighted (IVW) approach as the principal methodology. IVW was with multiplicative random effects whenever a minimum of four SNPs were present.^27^ When only two or three SNPs were available, a fixed‐effects IVW approach was used. To prevent the impact of undetectable and unknown confounders, supplementary analyses, including MR‐Egger,^28^ weighted median,^29^ weighted mode,^30^ and simple mode,^30^ were conducted. All analyses underwent adjustment through the Bonferroni method.^31^ In order to tackle multiple testing concerns, we adopted a conservative Bonferroni‐corrected *p*‐value <0.003 (0.05/15) via the IVW method to suggest statistical significance, as we examined the relationship between three HDPs‐associated traits and five gynecologic tumors. Results with *p*‐value >0.003 but less than 0.05 were considered as potential causal relationships.^32^, ^33^ To adjust for potential confounders, we executed multivariable MR (MVMR) analysis by incorporating SBP, BMI, and T2DM. The multivariable IVW, multivariable MR‐Egger, and multivariable median methods were used.^34^

### 2.4 Sensitivity analyses

We used the Cochran *Q* statistic to estimate the heterogeneity in IVW analysis (*p* < 0.05 was considered significant). To validate the existence of horizontal pleiotropy and evaluate the potential pleiotropic impacts of genetic instruments on the outcomes, we used MR‐Egger regression (*p* < 0.05 was considered significant).^28^ MR pleiotropy residual sum and outlier (MR‐PRESSO) test provided an additional sensitivity assessment. This method allowed the identification and removal of outliers in the MR summary data related to horizontal pleiotropy, and corrected horizontal pleiotropy. Furthermore, MR‐PRESSO test enabled the comparison of MR analysis results pre‐ and post‐correction (*p* < 0.05 was considered significant).^35^ For sensitivity analysis, we used the leave‐one‐out method to determine the impact of single SNPs on the overall findings. All statistical analyses were performed in R software 4.3.1 using the “TwoSampleMR”, “MVMR”, and “MR‐PRESSO” packages.

## 3 RESULTS

### 3.1 HDPs and common gynecologic tumors

**FIGURE 2:**
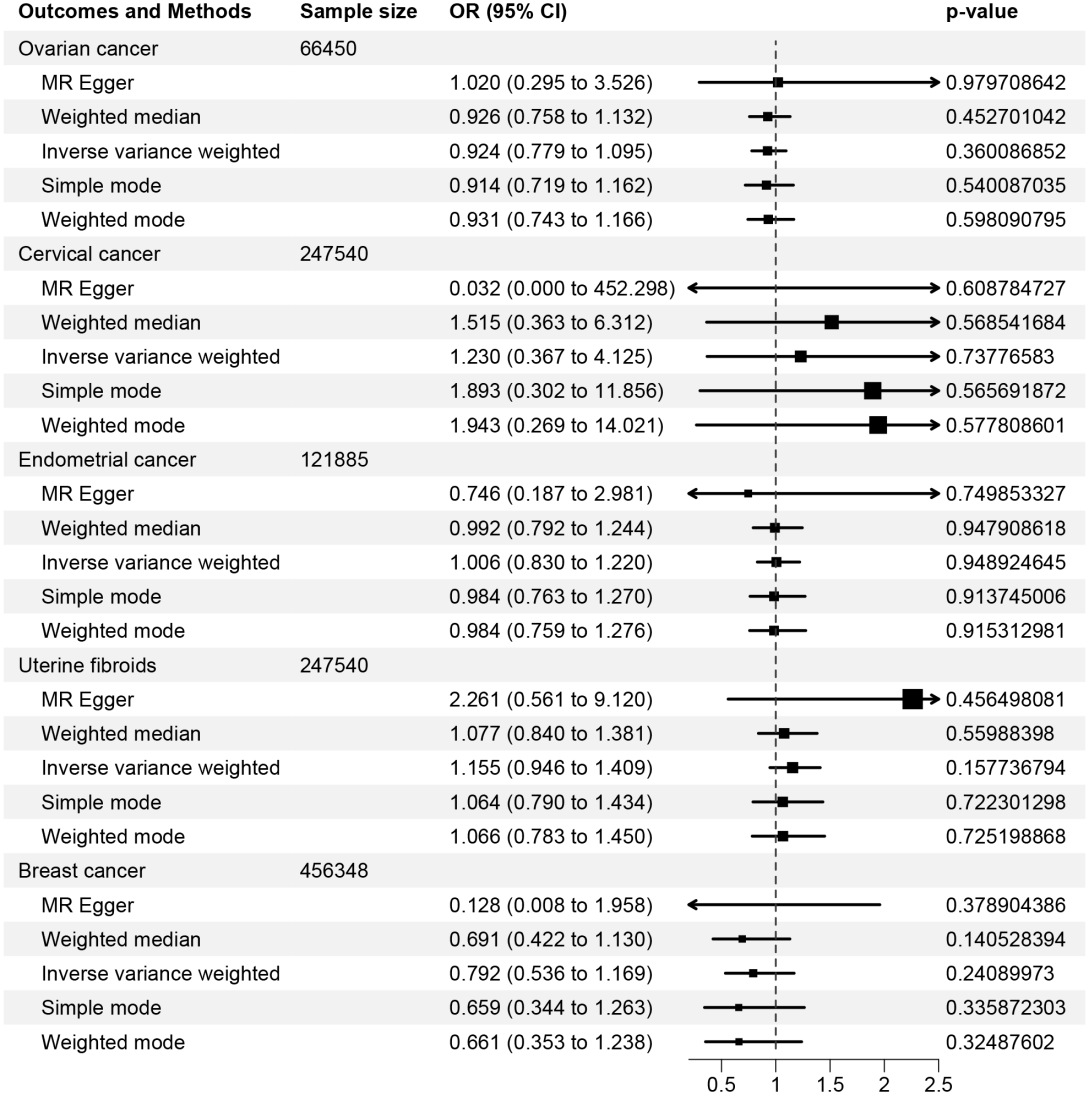
Causal association between any hypertensive disorders of pregnancy (HDPs) and common gynecologic tumors.

In the UVMR analysis, three SNPs were obtained from GWAS summary data of HDPs after excluding palindromic SNPs. No significant association has been observed between HDPs and the risks of ovarian cancer (OR [95% CI] = 0.924 [0.779–1.095]; *p* = 0.360), cervical cancer (OR [95% CI] = 1.230 [0.367–4.125]; *p* = 0.738), endometrial cancer (OR [95% CI] = 1.006 [0.830–1.220]; *p* = 0.949), uterine fibroids (OR [95% CI] = 1.155 [0.946–1.409]; *p* = 0.158), and breast cancer (OR [95% CI] = 0.792 [0.536–1.169]; *p* = 0.241) based on the primary causal effects model of IVW method. Similar results were also obtained through MR‐Egger, weighted median, weighted model and simple model methods (all *p* > 0.05) (Figure 2). No significant heterogeneity or horizontal pleiotropy was detected. The scatter plots of were provided in Figure S1. The leave‐one‐out sensitivity analysis revealed that the overall results were not affected by any individual SNP (Figure S2). MR‐PRESSO test was not conducted as it necessitates more than three SNPs for analysis (Table 2).

**TABLE 2:** Univariate Mendelian randomization (UVMR) analysis of hypertensive disorders of pregnancy (HDPs) with common gynecologic tumors in European population.

Outcomes	Methods	Beta	SE	*p*	Number of SNPs	*p* (Cochran's *Q* heterogeneity test)	*p* (MR‐Egger intercept test)	*p* (MR‐PRESSO global test)
Ovarian cancer	IVW	−0.079	0.087	0.360	3	0.904	0.900	NA
	MR‐Egger	0.020	0.633	0.980				
	Weighted median	−0.077	0.102	0.453				
	Simple mode	−0.090	0.123	0.540				
	Weighted mode	−0.071	0.115	0.598				
Cervical cancer	IVW	0.207	0.618	0.738	3	0.255	0.588	NA
	MR‐Egger	−3.440	4.875	0.609				
	Weighted median	0.415	0.728	0.569				
	Simple mode	0.638	0.936	0.566				
	Weighted mode	0.664	1.008	0.578				
Endometrial cancer	IVW	0.006	0.098	0.949	3	0.907	0.743	NA
	MR‐Egger	−0.293	0.707	0.750				
	Weighted median	−0.008	0.115	0.948				
	Simple mode	−0.016	0.130	0.914				
	Weighted mode	−0.016	0.133	0.915				
Uterine fibroids	IVW	0.144	0.102	0.158	3	0.544	0.515	NA
	MR‐Egger	0.816	0.711	0.456				
	Weighted median	0.074	0.127	0.56				
	Simple mode	0.062	0.152	0.722				
	Weighted mode	0.063	0.157	0.725				
Breast cancer	IVW	−0.233	0.199	0.241	3	0.372	0.412	NA
	MR‐Egger	−2.055	1.391	0.379				
	Weighted median	−0.37	0.251	0.141				
	Simple mode	−0.417	0.332	0.336				
	Weighted mode	−0.415	0.32	0.325				

In the MVMR analysis, the multivariable IVW method suggested that HDPs showed no significant association with ovarian cancer, cervical cancer, endometrial cancer, uterine fibroids, and breast cancer after adjusting for SBP, BMI, and T2DM (all *p* > 0.05). Consistent results were obtained from MVMR MR‐Egger method. However, the MVMR median method showed a potential relationship between HDPs and ovarian cancer (OR [95% CI] = 0.893 [0.805–0.991]; *p* = 0.033) after adjusting for SBP, and cervical cancer (OR [95% CI] = 1.986 [1.084–3.639]; *p* = 0.027) after adjusting for BMI. The MVMR results were detailed in Table 3.

**TABLE 3:** Multivariable Mendelian randomization (MVMR) analysis of hypertensive disorders of pregnancy with common gynecologic tumors in European population.

Outcomes	Methods	Adjustments	nSNP	OR	95% CI	*p* value
Ovarian cancer	Inverse variance weighted	SBP	350	0.908	0.802–1.028	0.092
		BMI	408	0.941	0.872–1.016	0.114
		T2DM	74	0.950	0.835–1.082	0.437
	MR Egger	SBP	350	0.882	0.775–1.004	0.059
		BMI	408	0.939	0.838–1.052	0.278
		T2DM	74	1.024	0.855–1.227	0.791
	MVMR median	SBP	350	0.893	0.805–0.991	0.033
		BMI	408	0.969	0.883–1.062	0.491
		T2DM	74	0.963	0.812–1.142	0.664
Cervical cancer	Inverse variance weighted	SBP	362	1.100	0.639–1.893	0.732
		BMI	419	1.330	0.816–2.166	0.251
		T2DM	77	0.977	0.390–2.450	0.962
	MR Egger	SBP	362	1.289	0.599–2.774	0.516
		BMI	419	1.029	0.498–2.126	0.937
		T2DM	77	0.578	0.159–2.101	0.404
	MVMR median	SBP	362	0.879	0.452–1.708	0.704
		BMI	419	1.986	1.084–3.639	0.027
		T2DM	77	2.219	0.660–7.465	0.198
Endometrial cancer	Inverse variance weighted	SBP	367	0.994	0.891–1.109	0.921
		BMI	426	1.012	0.925–1.108	0.790
		T2DM	78	1.075	0.848–1.362	0.552
	MR Egger	SBP	367	1.046	0.896–1.221	0.568
		BMI	426	0.931	0.813–1.065	0.293
		T2DM	78	1.055	0.755–1.476	0.753
	MVMR median	SBP	367	1.040	0.921–1.174	0.528
		BMI	426	1.044	0.941–1.158	0.421
		T2DM	78	1.134	0.932–1.380	0.206
Uterine fibroids	Inverse variance weighted	SBP	362	1.020	0.911–1.143	0.731
		BMI	419	0.992	0.898–1.096	0.877
		T2DM	77	1.146	0.927–1.416	0.207
	MR Egger	SBP	362	1.073	0.915–1.257	0.393
		BMI	419	0.901	0.775–1.048	0.175
		T2DM	77	1.089	0.808–1.467	0.579
	MVMR median	SBP	362	1.065	0.936–1.212	0.337
		BMI	419	1.037	0.922–1.166	0.551
		T2DM	77	1.112	0.896–1.379	0.338
Breast cancer	Inverse variance weighted	SBP	362	1.016	0.822–1.256	0.884
		BMI	419	1.051	0.864–1.279	0.616
		T2DM	77	0.846	0.599–1.195	0.340
	MR Egger	SBP	362	0.808	0.601–1.086	0.159
		BMI	419	1.058	0.791–1.414	0.704
		T2DM	77	0.753	0.463–1.224	0.252
	MVMR median	SBP	362	0.889	0.693–1.140	0.355
		BMI	419	0.969	0.773–1.213	0.779
		T2DM	77	0.705	0.444–1.119	0.137

Abbreviations: BMI, body mass index; HDPs, hypertensive disorders of pregnancy; IVW, inverse variance weighted; SBP, systolic blood pressure; T2DM, type 2 diabetes mellitus.

### 3.2 Gestational hypertension and common gynecologic tumors

**FIGURE 3:**
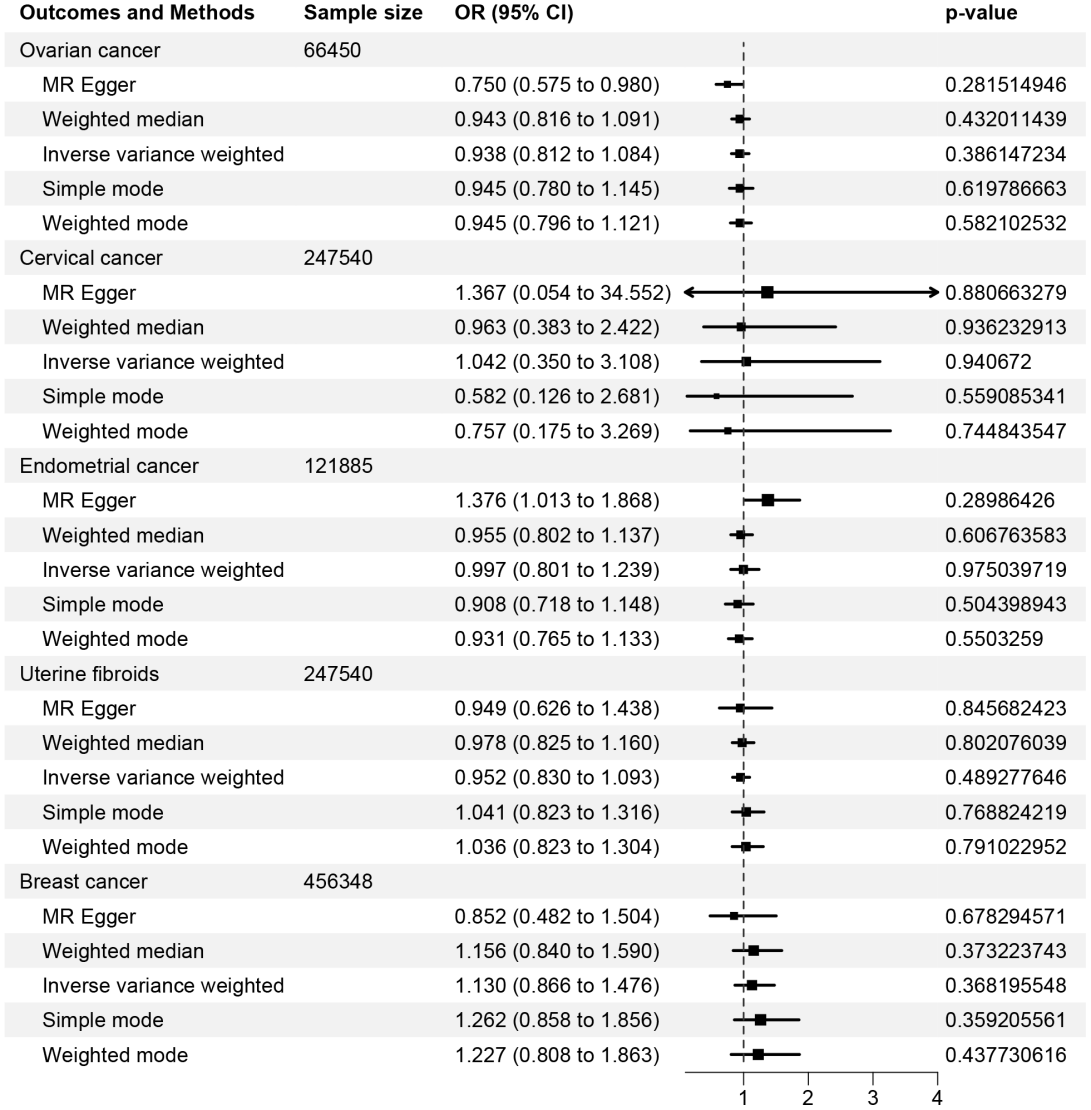
Causal association between gestational hypertension and common gynecologic tumors.

As shown in Figure 3; Tables 3 and 4 SNPs were ultimately obtained as the IVs for gestational hypertension to assess the associations with common gynecologic tumors. Genetically predicted gestational hypertension showed no association with ovarian cancer (OR [95% CI] = 0.938 [0.812–1.084]; *p* = 0.386), cervical cancer (OR [95% CI] = 1.042 [0.350–3.108]; *p* = 0.941), endometrial cancer (OR [95% CI] = 0.997 [0.801–1.239]; *p* = 0.975), uterine fibroids (OR [95% CI] = 0.952 [0.830–1.093]; *p* = 0.489), and breast cancer (OR [95% CI] = 1.130 [0.866–1.476]; *p* = 0.368). Consistent results were obtained from MR‐Egger, weighted median, weighted model, and simple model methods (all *p* > 0.05). Heterogeneity tests substantiated the absence of significant heterogeneity, validating the robustness of the results. Moreover, the MR‐Egger intercept did not suggest any evidence of pleiotropy. The scatter plots were shown in Figure S3. Leave‐one‐out analyses indicated that the exclusion of each SNP did not significantly impact the overall causal effect estimates, suggesting that no individual SNP had a significant influence on the overall results (Figure S4). The MR‐PRESSO test results were not available.

**TABLE 4:** Univariate Mendelian randomization (UVMR) analysis of gestational hypertension with common gynecologic tumors in European population.

Outcomes	Methods	Beta	SE	*p*	Number of SNPs	*p* (Cochran's *Q* heterogeneity test)	*p* (MR‐Egger intercept test)	*p* (MR‐PRESSO global test)
Ovarian cancer	IVW	−0.064	0.074	0.386	3	0.194	0.321	NA
	MR‐Egger	−0.287	0.136	0.282				
	Weighted median	−0.058	0.074	0.432				
	Simple mode	−0.057	0.098	0.620				
	Weighted mode	−0.057	0.088	0.582				
Cervical cancer	IVW	0.041	0.557	0.941	3	0.092	0.883	NA
	MR‐Egger	0.313	1.648	0.881				
	Weighted median	−0.038	0.471	0.936				
	Simple mode	−0.541	0.779	0.559				
	Weighted mode	−0.279	0.747	0.745				
Endometrial cancer	IVW	−0.003	0.111	0.975	3	0.063	0.263	NA
	MR‐Egger	0.319	0.156	0.290				
	Weighted median	−0.046	0.089	0.607				
	Simple mode	−0.097	0.120	0.504				
	Weighted mode	−0.071	0.100	0.550				
Uterine fibroids	IVW	−0.049	0.070	0.489	3	0.359	0.987	NA
	MR‐Egger	−0.052	0.212	0.846				
	Weighted median	−0.022	0.087	0.802				
	Simple mode	0.040	0.120	0.769				
	Weighted mode	0.035	0.117	0.791				
Breast cancer	IVW	0.123	0.136	0.368	3	0.527	0.468	NA
	MR‐Egger	−0.161	0.290	0.678				
	Weighted median	0.145	0.163	0.373				
	Simple mode	0.232	0.197	0.359				
	Weighted mode	0.205	0.213	0.438				

After adjusting for SBP, BMI, and T2DM, no significant correlation has been found between gestational hypertension and the risks of ovarian cancer, cervical cancer, endometrial cancer, uterine fibroids, and breast cancer based on the MVMR IVW method (all *p* > 0.05). Inconsistent results were obtained through MVMR MR‐Egger, and MVMR median methods. The MVMR MR‐Egger analysis showed a potential association between gestational hypertension and ovarian cancer (OR [95% CI] = 0.905 [0.819–1.000]; *p* = 0.049) after adjusting SBP. The MVMR median method found that after adjusting for SBP and T2DM, gestational hypertension was potentially associated with ovarian cancer (OR [95% CI] = 0.924 [0.856–0.997]; *p* = 0.043) and cervical cancer (OR [95% CI] = 2.423 [1.066–5.508]; *p* = 0.035), respectively (Table S4).

### 3.3 Preeclampsia/eclampsia and common gynecologic tumors

**FIGURE 4:**
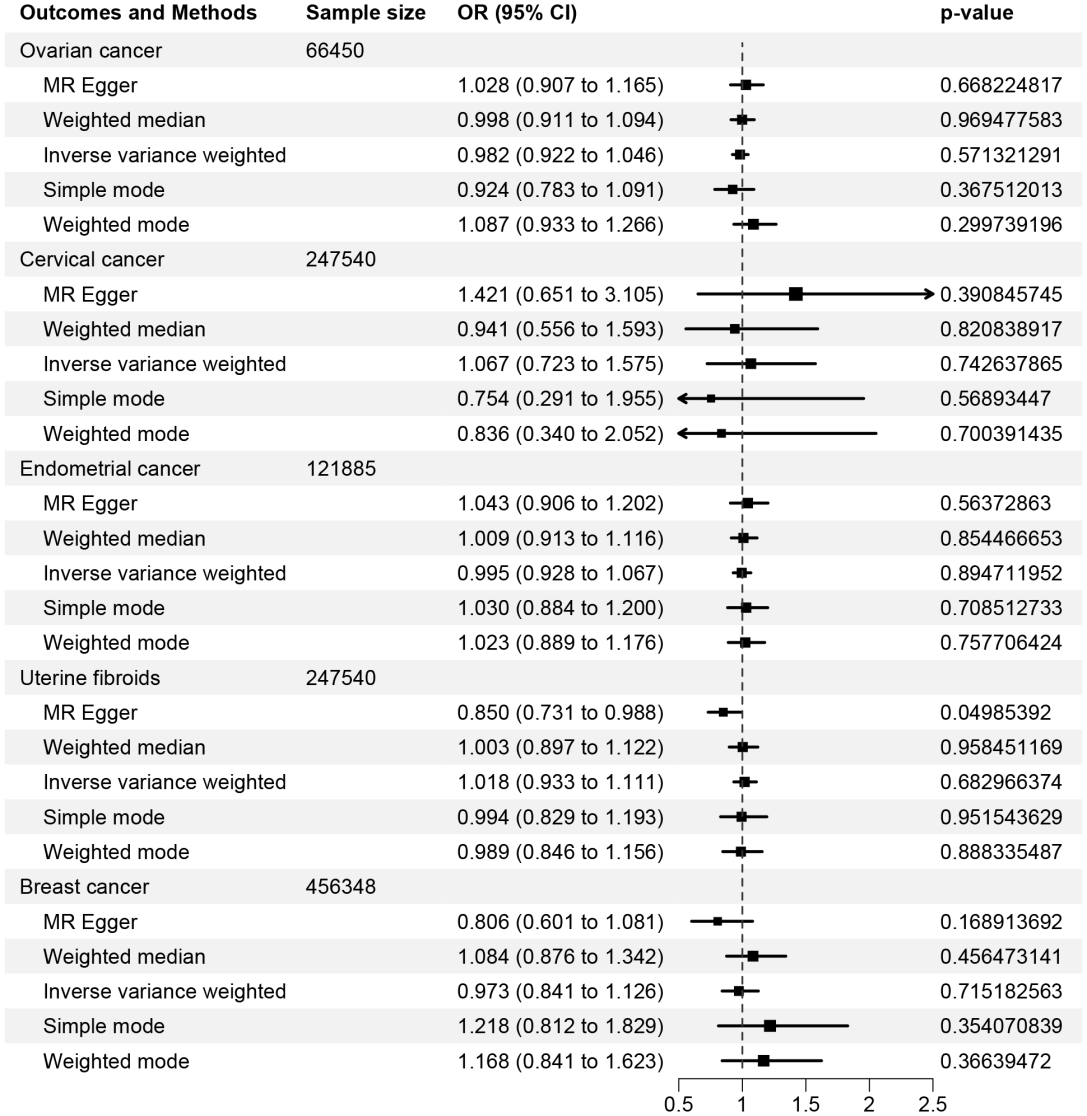
Causal association between preeclampsia/eclampsia and common gynecologic tumors.

In the UVMR analysis, 17, 18, 17, 18, and 18 SNPs were eventually obtained as the IVs for preeclampsia/eclampsia to evaluate the relationships with ovarian cancer, cervical cancer, endometrial cancer, uterine fibroids, and breast cancer, respectively. The IVW method indicated that pre‐eclampsia/eclampsia showed no significant correlation with ovarian cancer (OR [95% CI] = 0.982 [0.922–1.046]; *p* = 0.571), cervical cancer (OR [95% CI] = 1.067 [0.723–1.575]; *p* = 0.743), endometrial cancer (OR [95% CI] = 0.995 [0.928–1.067]; *p* = 0.895), uterine fibroids (OR [95% CI] = 1.018 [0.933–1.111]; *p* = 0.683), and breast cancer (OR [95% CI] = 0.973 [0.841–1.126]; *p* = 0.715). The weighted median, weighted model, and simple model results were consistent with IVW (all *p* > 0.05). While the MR‐Egger analysis showed a potential relationship between pre‐eclampsia/eclampsia and uterine fibroids (OR [95% CI] = 0.850 [0.731–0.988]; *p* = 0.050) (Figure 4). The absence of heterogeneity was confirmed through a heterogeneity analysis. MR‐Egger intercept test indicated the possibility of horizontal pleiotropy only in the outcome of uterine fibroids (*p* = 0.015). Nonetheless, after running 2000 simulations via the MR‐PRESSO method, no outliers or horizontal pleiotropy were detected (*p =* 0.151) (Table 5). This finding suggested that the IVs affected the uterine fibroids risk exclusively through their effect on pre‐eclampsia/eclampsia. The scatter plots were presented in Figure S5. Additionally, leave‐one‐out analysis also did not identify any abnormal SNPs (Figure S6).

**TABLE 5:** Univariate Mendelian randomization (UVMR) analysis of preeclampsia/eclampsia with common gynecologic tumors in European population.

Outcomes	Methods	Beta	SE	p	Number of SNPs	*p* (Cochran's *Q* heterogeneity test)	*p* (MR‐Egger intercept test)	*p* (MR‐PRESSO global test)
Ovarian cancer	IVW	−0.018	0.032	0.571	17	0.460	0.418	0.438
	MR‐Egger	0.028	0.064	0.668				
	Weighted median	−0.002	0.047	0.969				
	Simple mode	−0.079	0.085	0.368				
	Weighted mode	0.083	0.078	0.300				
Cervical cancer	IVW	0.065	0.198	0.743	18	0.910	0.420	0.909
	MR‐Egger	0.352	0.399	0.391				
	Weighted median	−0.061	0.269	0.821				
	Simple mode	−0.282	0.486	0.569				
	Weighted mode	−0.179	0.458	0.700				
Endometrial cancer	IVW	−0.005	0.036	0.895	17	0.622	0.463	0.637
	MR‐Egger	0.043	0.072	0.564				
	Weighted median	0.009	0.051	0.854				
	Simple mode	0.030	0.078	0.709				
	Weighted mode	0.022	0.071	0.758				
Uterine fibroids	IVW	0.018	0.045	0.683	18	0.144	0.015	0.151
	MR‐Egger	−0.163	0.077	0.050				
	Weighted median	0.003	0.057	0.958				
	Simple mode	−0.006	0.093	0.952				
	Weighted mode	−0.011	0.080	0.888				
Breast cancer	IVW	−0.027	0.075	0.715	18	0.561	0.166	0.564
	MR‐Egger	−0.216	0.150	0.169				
	Weighted median	0.081	0.109	0.456				
	Simple mode	0.197	0.207	0.354				
	Weighted mode	0.156	0.168	0.366				

MVMR analysis revealed that preeclampsia/eclampsia showed no significant association with ovarian cancer, cervical cancer, endometrial cancer, uterine fibroids, and breast cancer after adjusting for SBP, BMI, and T2DM (all *p* > 0.05). Similar results were obtained through MVMR MR‐Egger method. Nevertheless, the MVMR median method showed a potential association between preeclampsia/eclampsia and ovarian cancer (OR [95% CI] = 0.918 [0.855–0.985]; *p* = 0.018) after adjusting for SBP (Table S5).

## 4 DISCUSSION

By using genetic data, we explored the relationship of HDPs on risk of five common gynecologic tumors. The results revealed that there is no strong evidence to support causal association between HDPs and the risks of ovarian, cervical, endometrial, breast cancer, and uterine fibroids.

In the preceding decades, multiple studies have been carried out to explore the relationships between HDPs and gynecologic tumors, yielding inconsistent results due to variations in the study cohorts, research methodology, follow‐up duration, and adjustments for potential confounding variables.^11^, ^12^, ^13^, ^36^, ^37^ A longitudinal study, featuring an average follow‐up duration of 19 years, conclude that HDPs did not constitute an independent risk for prevalent female cancers (ovarian, endometrial, and breast).^11^ However, a nationwide cohort investigation with an average follow‐up period of 17.7 years discovered that a history of HDPs correlated with a diminished occurrence of breast cancer and an augmented incidence of endometrial cancer, but had no association with ovarian or cervical cancer.^12^ This MR analysis, using European ancestry data, determined that genetically predicted HDPs were not associated with gynecologic tumors. Nevertheless, further stratified analysis by race or country is challenging to achieve, which may contribute to the discrepancies between the MR analysis results and those of earlier investigations. A contemporary Nordic research, encompassing 116, 196 breast cancer cases, demonstrated a reduced risk of breast cancer in women with a history of gestational hypertension or preeclampsia, following adjustments for country, maternal birth year, and parity.^38^ Subsequently, an elevated endometrial cancer risk in Nordic population was observed to be associated with HDPs, including gestational hypertension and preeclampsia.^39^ Opposite findings regarding the reduction in cancer risk have been documented in studies conducted on populations from Sweden, Norway, Scotland, and the United States.^6^, ^7^, ^10^, ^40^, ^41^ Moreover, an Italian case–control research has identified a significant elevation in the risk of breast cancer, which was associated with a history of gestational hypertension.^42^ A recent nationwide prospective cohort study involving 40, 720 parous women found no associations between a history of HDPs and risk of breast cancer. However, when the analysis was stratified according to race and ethnicity, a positive association was suggested between HDPs and the risk of breast cancer among Hispanic/Latina women.^13^ These varying findings might be attributed to underlying differences in various populations. Consequently, it is plausible that intrinsic genetic diversity among different study populations could lead to disparate outcomes. Further MR analyses are warranted across diverse populations to better understand these relationships.

Most current studies tend to focus on HDPs, especially preeclampsia, and future risks of breast cancer and endometrial cancer. A 2021 meta‐analysis deduced that women who have experienced preeclampsia have a 19% reduced risk of developing premenopausal breast cancer.^43^ This inverse relationship was further substantiated by a combined analysis of six separate cohorts.^36^ Several theories have been put forth to clarify the observed decrease in breast cancer risk among individuals with a history of HDPs, specifically preeclampsia. The prevailing hypothesis suggests that preeclampsia/eclampsia incites hormonal upheavals and imbalances during pregnancy, which could potentially correlate with the ensuing risk of hormone‐dependent cancers.^44^ The anti‐angiogenic profile associated with preeclampsia/eclampsia might confer a protective effect against cancer, considering the necessity of angiogenesis for the growth and metastasis of tumors.^38^, ^45^ This suggests a potential correlation between preeclampsia/eclampsia and a diminished likelihood of gynecologic tumors later in life. Supporting this hypothesis, a decrease in breast cancer risk has been recorded in women who have a history of preeclampsia/eclampsia,^44^ particularly among premenopausal women and those with breast cancer associated with ERB‐B2 receptor tyrosine kinase 2.^6^, ^7^, ^46^ However, this association is not universally recognized. Research based on the Norfolk prospective population‐based study discovered no association between HDPs and breast cancer.^11^ Previous Finnish cohort research suggested that the incidence of breast cancer in postmenopausal hypertension patients does not seem to be higher than that in the general population.^47^ In addition, a recent Taiwanese study also failed to identify an association between HDPs and breast cancer risk, despite a point estimate leaning towards an increased risk.^48^ By using MR analysis, we can provide a genetic evidence that HDPs does not amplify the risk of subsequent breast cancer. Although our MR analysis was unaffected by confounders, GWAS summary data on breast cancer lacked information on cancer type and stage, which limited our further stratified exploration. Therefore, the present MR analysis cannot provide a comprehensive explanation for the relationship between HDPs and different subtypes and premenopausal or postmenopausal gynecologic tumors. Future research is warranted to further explore the relationships between HDPs and different types and stages of gynecologic tumors.

Analogous to breast cancer, the majority of endometrial cancers exhibit hormone‐dependency, with key risk factors linked to exposure to endogenous and/or exogenous estrogens.^49^ Women with preeclampsia tend to exhibit lower estrogen levels and heightened progesterone levels relative to those with normotensive pregnancies.^50^ Additionally, preeclamptic women may exhibit increased androgen levels, potentially attributable to inadequate placental enzyme production for the aromatization of testosterone to estrogen and elevated inhibin A levels, leading to augmented androgen production.^51^ Several large prospective studies have suggested a correlation between augmented circulating testosterone concentrations or genetic indicators suggestive of elevated testosterone levels, and a heightened risk of endometrial cancer.^52^, ^53^ Furthermore, the elevated risks related to hypertension and preeclampsia indicated that immunological and inflammatory causes during and prior to pregnancy may also be significant contributors to developing endometrial cancer. Specifically, a connection has been established between preeclampsia and the augmentation of inflammation and immune activation in maternal circulation and the uteroplacental unit,^54^ factors that could be relevant to the escalated risk of endometrial cancer. However, epidemiological studies examining the association between preeclampsia and endometrial cancer risk have yielded inconsistent results. For instance, a large Danish case–control research discovered no correlation between preeclampsia and the risk of endometrial cancer. However, a stratified analysis revealed an elevated risk of endometrial cancer in cases of early onset preeclampsia, while no correlation was observed in instances of late onset disease.^55^ The Jerusalem Perinatal Study found no correlation between preeclampsia and uterine cancer.^56^ While the Jerusalem Perinatal Study did not acquire data pertaining to gestational age, it remains conceivable that a significant number of preeclampsia diagnoses were of late onset. Conversely, a nested case–control study encompassing 10, 924 endometrial cancer instances from four Nordic countries concluded that preeclampsia during pregnancy was markedly linked to an increased risk of endometrial cancer. This correlation was observed similarly for both Type I and Type II endometrial cancer.^39^ Unlike previous observational studies with inconsistent results, this MR analysis provides reliable evidence to support no association between HDPs and future endometrial cancer risk. Early‐ and late‐onset preeclampsia may have different effects on endometrial cancer. Further exploring the association between the subtypes of preeclampsia/eclampsia or gestational hypertension and gynecologic tumors is needed.

Few studies reported the association between HDPs and the risks of ovarian and cervical cancer, as well as uterine fibroids. A cohort study using Swedish cancer registry data found no correlation between preeclampsia and cervical cancer.^57^ Similarly, a prospective population‐based investigation with an average follow‐up of 19 years determined no connection between HDPs and ovarian cancer.^11^ A comprehensive meta‐analysis, aggregating 13 studies and incorporating 5, 254, 150 participants, suggested no substantial disparity in the susceptibility to uterus‐associated malignancies between women experiencing normal pregnancies and those afflicted with preeclampsia. However, the peril of ovarian cancer was amplified in women experiencing preeclampsia relative to those undergoing normal pregnancies.^43^ Prior researches have merely suggested a correlation between hypertension and uterine fibroids.^58^, ^59^ At the commencement of hypertension, angiotensin undergoes hydrolysis to form angiotensin I, which is subsequently transformed into angiotensin II via the action of the angiotensin converting enzyme.^60^ Research has indicated that angiotensin II notably escalates the population of uterine leiomyoma cells, with this effect being proportionate to the dosage.^61^ A study conducted by Hsieh et al. discovered a significant correlation between mutations in genes responsible for activating angiotensin‐converting enzyme and susceptibility to leiomyoma.^62^ These findings suggested that the production of angiotensin II, as a result of hypertension, could potentially trigger uterine leiomyoma. Further, hypertension may stimulate the proliferation of fibroids and fibrogenesis by causing injury to smooth muscle cells via mechanical shear stress, which could also contribute to the development of uterine fibroids.^63^ Nevertheless, there are currently no reports on the connection between HDPs and the subsequent risk of developing uterine fibroids. The recent MR study have shown that higher genetically predicted SBP and diastolic blood pressure (DBP) were related to an increased risk of uterine fibroids.^64^ To our knowledge, this is the first MR study to report the association between HDPs and ovarian cancer, cervical cancer, and uterine fibroids. The genetic prediction that HDPs are not associated with ovarian cancer, cervical cancer, and uterine fibroids may provide valuable insights for future research directions: (1) population‐ or MR‐based studies are needed to further explore the association of HDPs with ovarian cancer, cervical cancer, and uterine fibroids in non‐European populations, such as East Asian and African populations; (2) the association between HDPs and subtypes and stages of various gynecologic tumors needs further research and validation.

However, several limitations within this study are worth considering. First, our findings are derived from data of European ancestry in an effort to control for racial effects, which consequently limits their applicability to non‐European populations. Second, potential sample overlap may exist given that both exposure and outcome datasets originate from European populations. Unfortunately, the evaluation of overlapping sample sizes continues to present a challenge. The presence of overlapping samples may bias the estimations derived from a two‐sample MR study. Recent research posited that two‐sample MR approach can be reliably employed when dealing with large data from a single source like UK Biobank.^65^ Although our findings indicated no association between HDPs and common gynecologic tumors, it needs to be verified in further MR studies. Third, the restriction of GWAS summary data on HDPs and gynecologic tumors makes it challenging to stratify research based on the current results, an issue that warrants attention upon future dataset updates.

## 5 CONCLUSION

In conclusion, this MR study did not find causal effects of HDPs on ovarian, cervical, endometrial, breast cancer, and uterine fibroids. Further exploration of the impact of HDPs on the subtypes of gynecologic tumors in different ethnic populations is needed.

## AUTHOR CONTRIBUTIONS

**Le Zhou:** Formal analysis (lead); investigation (lead); software (equal); writing – original draft (lead). **Xinghui Liu:** Data curation (lead); methodology (lead); visualization (lead); writing – review and editing (equal). **Guolin He:** Formal analysis (equal); software (equal); writing – review and editing (equal). **Chuntang Sun:** Conceptualization (lead); supervision (lead); writing – review and editing (equal).

## CONFLICT OF INTEREST STATEMENT

The authors have no conflicts of interest to disclose.

## ETHICS STATEMENT

Additional ethical approval was not required as we used published data for re‐analysis.

## Supporting information


Figure S1:

Table S1:

## Data Availability

All data included in this study are publicly available GWAS summary data.
