# Supplementary material for: Causal effects of hypertensive disorders of pregnancy on future gynecologic tumors: A two‐sample Mendelian randomization study
Source: Cancer Med. 2024 May 27;13(10):e7300. doi: 10.1002/cam4.7300 (PMC11129165; doi:10.1002/cam4.7300)
Supplement: Supplementary file 1 — Figure S1: [file CAM4-13-e7300-s001.pdf]

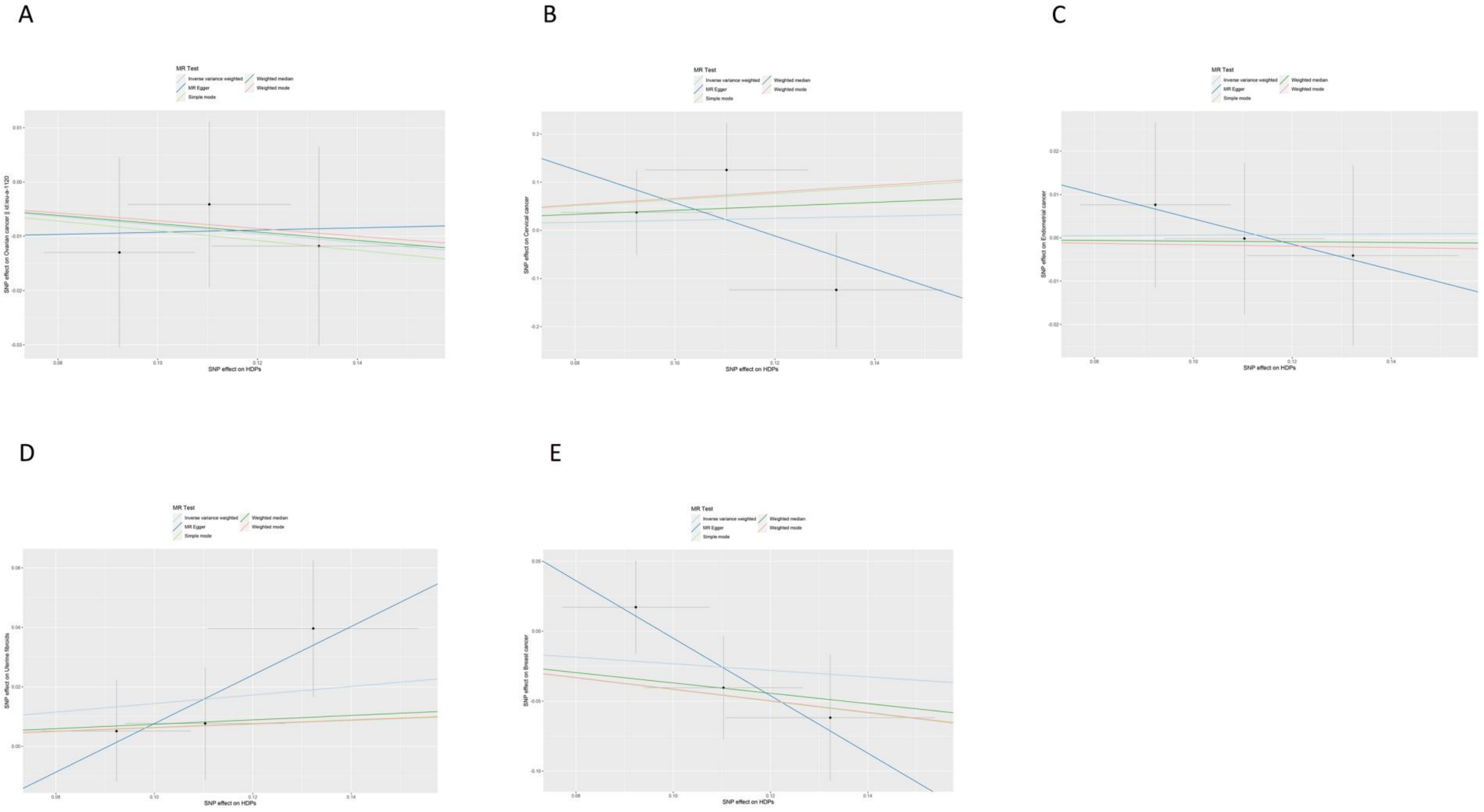

FIGURE S1 Scatter plots of any hypertensive disorders of pregnancy on common gynecologic tumors. (A) Ovarian cancer; (B) Cervical cancer; (C) Endometrial cancer; (D) Uterine fibroids; (E) Breast cancer.

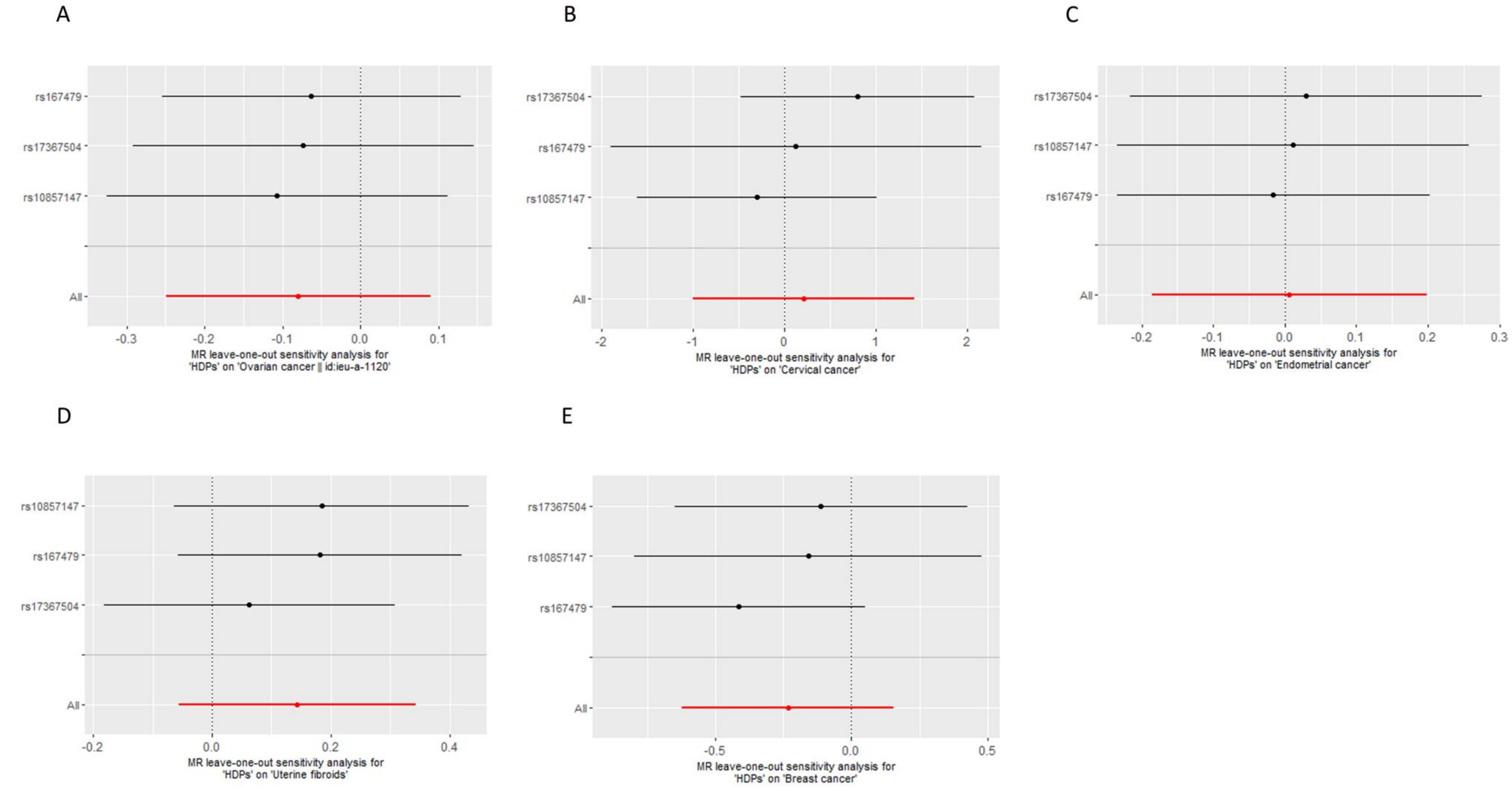

FIGURE S2 Leave-one-out analyses of any hypertensive disorders of pregnancy on common gynecologic tumors. (A) Ovarian cancer; (B) Cervical cancer; (C) Endometrial cancer; (D) Uterine fibroids; (E) Breast cancer.

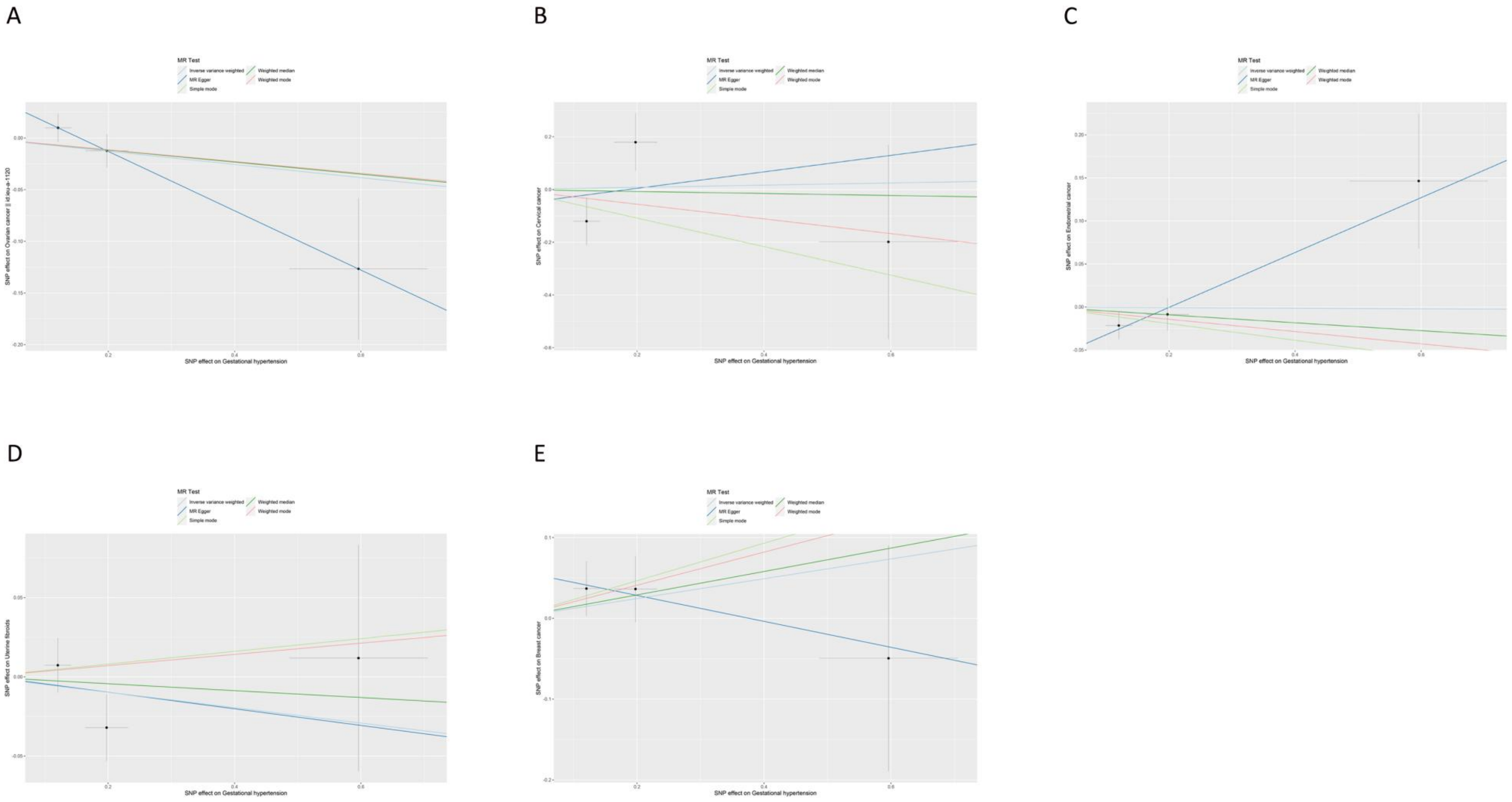

FIGURE S3 Scatter plots of gestational hypertension on common gynecologic tumors. (A) Ovarian cancer; (B) Cervical cancer; (C) Endometrial cancer; (D) Uterine fibroids; (E) Breast cancer.

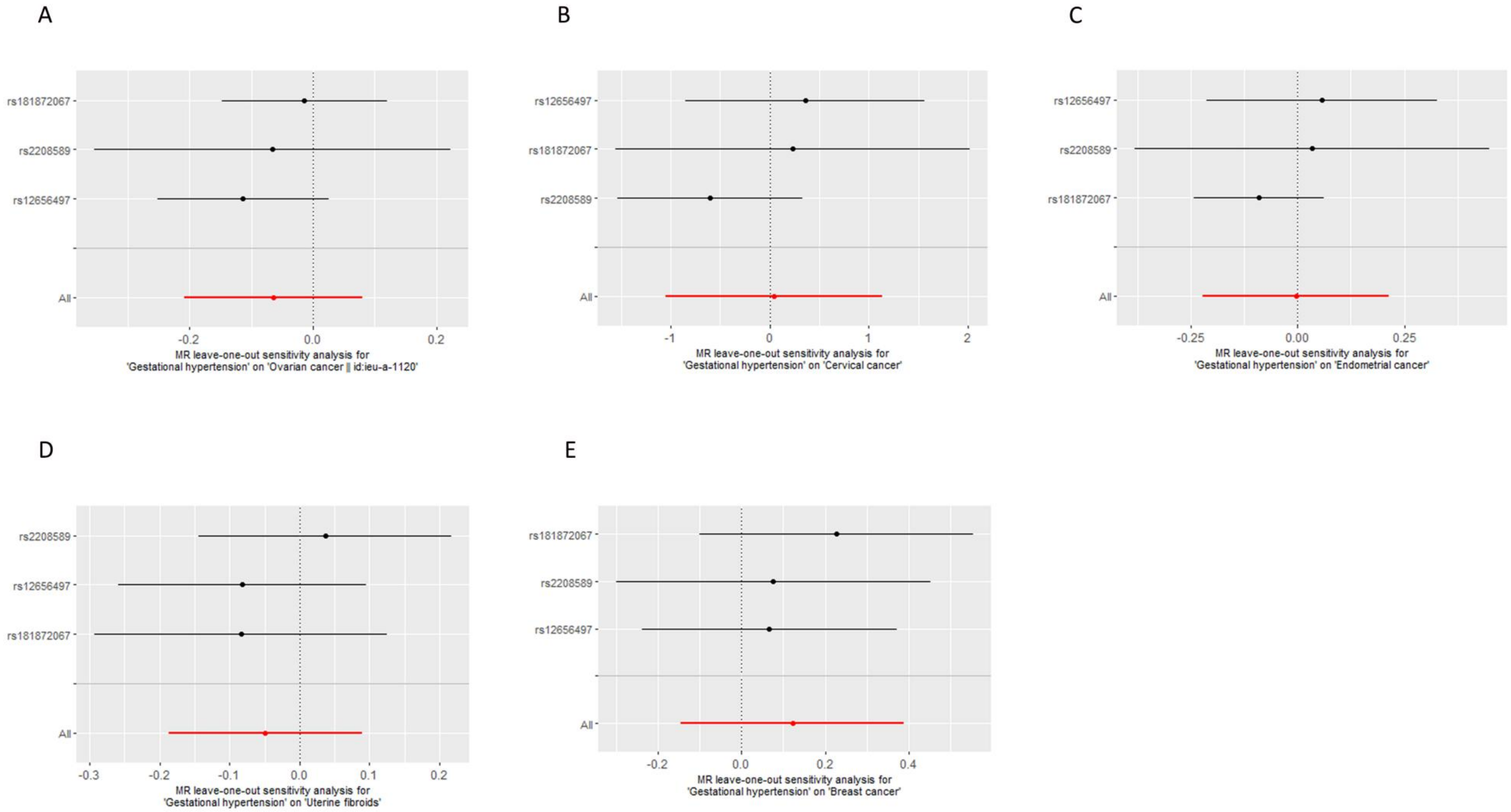

FIGURE S4 Leave-one-out analyses of gestational hypertension on common gynecologic tumors. (A) Ovarian cancer; (B) Cervical cancer; (C) Endometrial cancer; (D) Uterine fibroids; (E) Breast cancer.

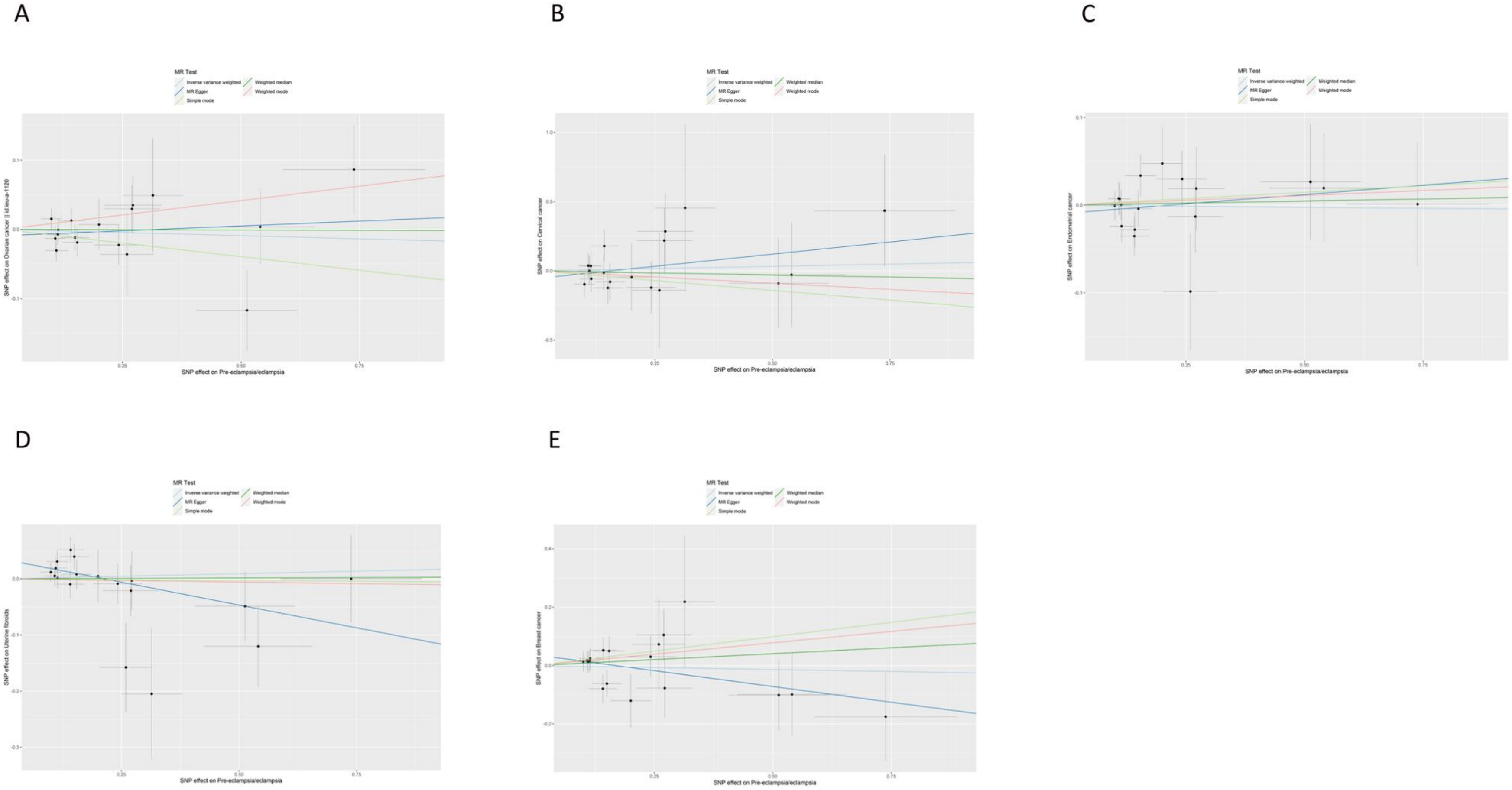

FIGURE S5 Scatter plots of preeclampsia/eclampsia on common gynecologic tumors. (A) Ovarian cancer; (B) Cervical cancer; (C) Endometrial cancer; (D) Uterine fibroids; (E) Breast cancer.

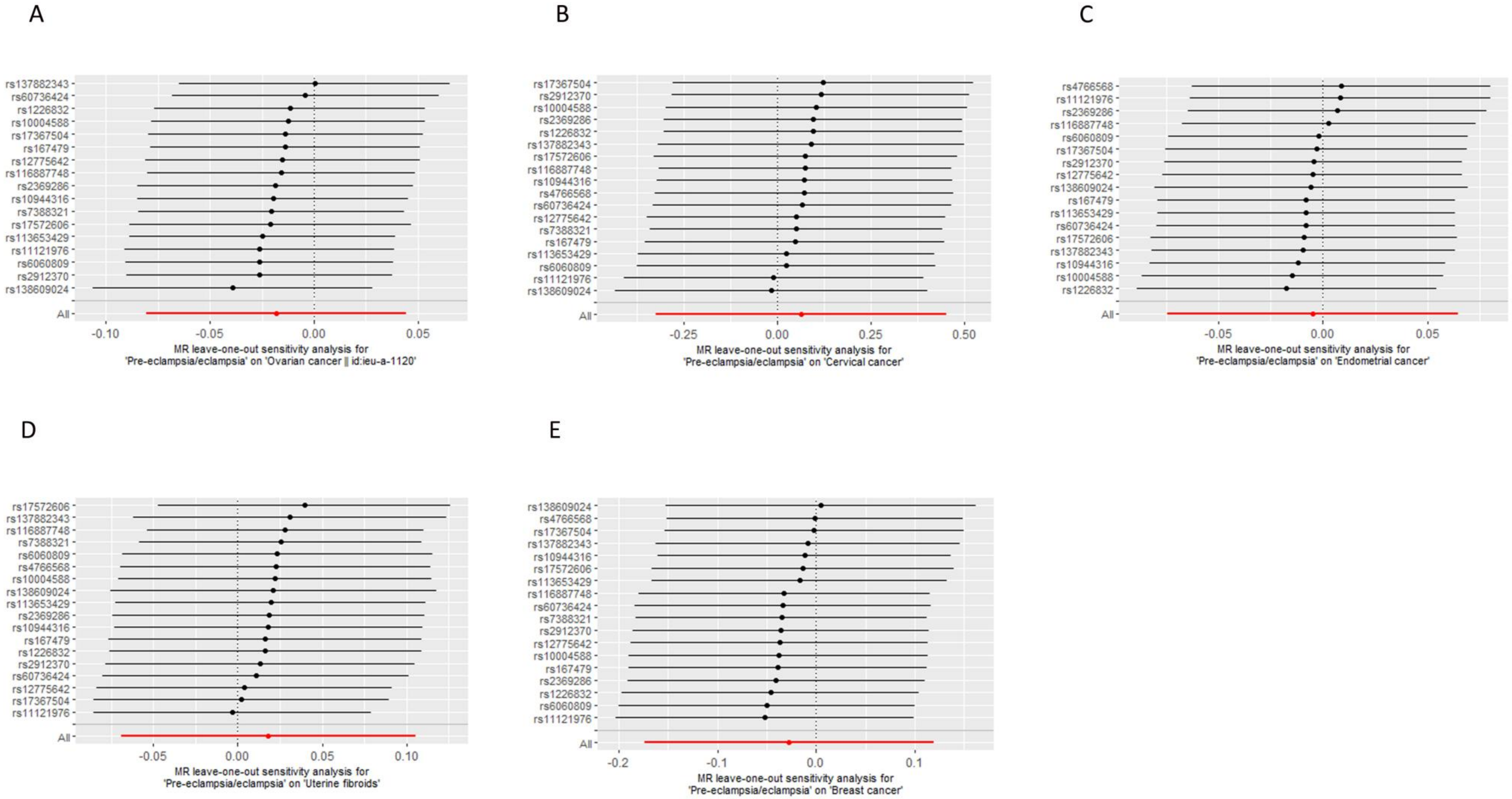

FIGURE S6 Leave-one-out analyses of preeclampsia/eclampsia on common gynecologic tumors. (A) Ovarian cancer; (B) Cervical cancer; (C) Endometrial cancer; (D) Uterine fibroids; (E) Breast cancer.
