# Supplementary material for: Causal effects of hypertensive disorders of pregnancy on future gynecologic tumors: A two‐sample Mendelian randomization study
Source: Cancer Med. 2024 May 27;13(10):e7300. doi: 10.1002/cam4.7300 (PMC11129165; doi:10.1002/cam4.7300)
Supplement: Supplementary file 2 — Table S1: [file CAM4-13-e7300-s002.docx]

| **TABLE S1** List of instrumental variables used in the analyses for the exposure of hypertensive disorders of pregnancy, and corresponding gene-outcome association estimates for all SNPs available in outcome GWAS. | | | | | | | | | | |
| --- | --- | --- | --- | --- | --- | --- | --- | --- | --- | --- |
| **Hypertensive disorders of pregnancy** | | | | | | | | | | |
| SNP | effect_allele | other_allele | eaf | beta | se | pval | R^2^ | F |  |  |
| rs17367504 | G | A | 0.144 | -0.13229 | 0.02144 | 6.77E-10 | 0.000258818 | 38.0713752 |  |  |
| rs10857147 | T | A | 0.313 | 0.11033 | 0.01627 | 1.20E-11 | 0.000312593 | 45.98394469 |  |  |
| rs10882398 | A | T | 0.594 | 0.08859 | 0.01529 | 6.81E-09 | 0.000228222 | 33.5697824 |  |  |
| rs167479 | G | T | 0.574 | 0.09232 | 0.01518 | 1.19E-09 | 0.000251444 | 36.98640681 |  |  |
| **Ovarian cancer** | | | | | | | | | | |
| SNP | effect_allele | other_allele | palindromic | ambiguous | chr | pos | eaf | beta | se | pval |
| rs10857147 | T | A | TRUE | FALSE | 4 | 81181072 | 0.3077 | -0.004114 | 0.01524 | 0.7872 |
| rs10882398 | A | T | TRUE | TRUE | 10 | 95892788 | 0.542 | -0.02103 | 0.01341 | 0.1169 |
| rs167479 | G | T | FALSE | FALSE | 19 | 11526765 | 0.505 | -0.01297 | 0.01748 | 0.457999 |
| rs17367504 | G | A | FALSE | FALSE | 1 | 11862778 | 0.1662 | 0.0118 | 0.01829 | 0.5187 |
| **Cervical cancer** | | | | | | | | | | |
| SNP | effect_allele | other_allele | palindromic | ambiguous | chr | pos | eaf | beta | se | pval |
| rs10857147 | T | A | TRUE | FALSE | 4 | 81181072 | 0.289235 | 0.125336 | 0.0978311 | 0.200142 |
| rs10882398 | A | T | TRUE | TRUE | 10 | 95892788 | 0.566268 | 0.12512 | 0.089597 | 0.162571 |
| rs167479 | G | T | FALSE | FALSE | 19 | 11526765 | 0.528339 | 0.0371751 | 0.088344 | 0.673901 |
| rs17367504 | G | A | FALSE | FALSE | 1 | 11862778 | 0.162156 | 0.123774 | 0.119679 | 0.301035 |
| **Endometrial cancer** | | | | | | | | | | |
| SNP | effect_allele | other_allele | palindromic | ambiguous | chr | pos | eaf | beta | se | pval |
| rs10857147 | T | A | TRUE | FALSE | 4 | 81181072 | 0.302077 | -0.000161046 | 0.0174254 | 0.992626 |
| rs10882398 | A | T | TRUE | TRUE | 10 | 95892788 | 0.556037625 | 0.000375398 | 0.0154299 | 0.98059 |
| rs167479 | G | T | FALSE | FALSE | 19 | 11526765 | 0.520121625 | 0.00762732 | 0.0190058 | 0.688188 |
| rs17367504 | G | A | FALSE | FALSE | 1 | 11862778 | 0.1564785 | 0.00409044 | 0.0209032 | 0.844857 |
| **Uterine fibroids** | | | | | | | | | | |
| SNP | effect_allele | other_allele | palindromic | ambiguous | chr | pos | eaf | beta | se | pval |
| rs10857147 | T | A | TRUE | FALSE | 4 | 81181072 | 0.289235 | 0.00767862 | 0.0188494 | 0.683738 |
| rs10882398 | A | T | TRUE | TRUE | 10 | 95892788 | 0.566268 | 0.012629 | 0.0172641 | 0.464464 |
| rs167479 | G | T | FALSE | FALSE | 19 | 11526765 | 0.528339 | 0.00511119 | 0.0170207 | 0.763954 |
| rs17367504 | G | A | FALSE | FALSE | 1 | 11862778 | 0.162156 | -0.03961 | 0.0230337 | 0.0854944 |
| **Breast cancer** | | | | | | | | | | |
| SNP | effect_allele | other_allele | palindromic | ambiguous | chr | pos | eaf | beta | se | pval |
| rs10857147 | T | A | TRUE | FALSE | 4 | 81181072 | 0.289415 | -0.0404177 | 0.0368245 | 0.272389 |
| rs10882398 | A | T | TRUE | TRUE | 10 | 95892788 | 0.565966 | -0.0568514 | 0.0337954 | 0.0925257 |
| rs167479 | G | T | FALSE | FALSE | 19 | 11526765 | 0.528524 | 0.0171364 | 0.0333076 | 0.60691 |
| rs17367504 | G | A | FALSE | FALSE | 1 | 11862778 | 0.162091 | 0.0618116 | 0.0450113 | 0.169676 |
| SNP, single nucleotide polymorphism; eaf, effect allele frequency; se, standard error; pval, p value; F, F-statistic; chr, chromosome; pos, position. | | | | | | | | | | |

| **TABLE S2** List of instrumental variables used in the analyses for the exposure of gestational hypertension, and corresponding gene-outcome association estimates for all SNPs available in outcome GWAS. | | | | | | | | | | |
| --- | --- | --- | --- | --- | --- | --- | --- | --- | --- | --- |
| **Gestational hypertension** | | | | | | | | | | |
| SNP | effect_allele | other_allele | eaf | beta | se | pval | R^2^ | F |  |  |
| rs181872067 | A | G | 0.0103 | 0.59612 | 0.10925 | 4.86E-08 | 0.00021027 | 29.77270941 |  |  |
| rs12656497 | C | T | 0.586 | 0.12036 | 0.02112 | 1.21E-08 | 0.000229362 | 32.47658792 |  |  |
| rs2208589 | G | A | 0.888 | 0.19762 | 0.03403 | 6.33E-09 | 0.000238165 | 33.72343123 |  |  |
| **Ovarian cancer** | | | | | | | | | | |
| SNP | effect_allele | other_allele | palindromic | ambiguous | chr | pos | eaf | beta | se | pval |
| rs12656497 | C | T | FALSE | FALSE | 5 | 32831939 | 0.5864 | 0.009874 | 0.01383 | 0.4753 |
| rs181872067 | A | G | FALSE | FALSE | 2 | 8825572 | 0.0129 | -0.1266 | 0.06847 | 0.0644006 |
| rs2208589 | G | A | FALSE | FALSE | 20 | 47408414 | 0.777 | -0.01238 | 0.01613 | 0.4428 |
| **Cervical cancer** | | | | | | | | | | |
| SNP | effect_allele | other_allele | palindromic | ambiguous | chr | pos | eaf | beta | se | pval |
| rs12656497 | C | T | FALSE | FALSE | 5 | 32831939 | 0.597012 | -0.120297 | 0.0898755 | 0.180738 |
| rs181872067 | A | G | FALSE | FALSE | 2 | 8825572 | 0.0144668 | -0.198549 | 0.369241 | 0.590769 |
| rs2208589 | G | A | FALSE | FALSE | 20 | 47408414 | 0.78156 | 0.179475 | 0.10911 | 0.09999 |
| **Endometrial cancer** | | | | | | | | | | |
| SNP | effect_allele | other_allele | palindromic | ambiguous | chr | pos | eaf | beta | se | pval |
| rs12656497 | C | T | FALSE | FALSE | 5 | 32831939 | 0.59184575 | -0.021494 | 0.015898 | 0.176377 |
| rs181872067 | A | G | FALSE | FALSE | 2 | 8825572 | 0.011985625 | 0.146385 | 0.0784016 | 0.0618848 |
| rs2208589 | G | A | FALSE | FALSE | 20 | 47408414 | 0.779429125 | -0.00864632 | 0.018991 | 0.648904 |
| **Uterine fibroids** | | | | | | | | | | |
| SNP | effect_allele | other_allele | palindromic | ambiguous | chr | pos | eaf | beta | se | pval |
| rs12656497 | C | T | FALSE | FALSE | 5 | 32831939 | 0.597012 | 0.0073198 | 0.017315 | 0.672482 |
| rs181872067 | A | G | FALSE | FALSE | 2 | 8825572 | 0.0144668 | 0.0118314 | 0.0714149 | 0.868416 |
| rs2208589 | G | A | FALSE | FALSE | 20 | 47408414 | 0.78156 | -0.0320604 | 0.0209869 | 0.126601 |
| **Breast cancer** | | | | | | | | | | |
| SNP | effect_allele | other_allele | palindromic | ambiguous | chr | pos | eaf | beta | se | pval |
| rs12656497 | C | T | FALSE | FALSE | 5 | 32831939 | 0.596581 | 0.0368532 | 0.0338829 | 0.276744 |
| rs181872067 | A | G | FALSE | FALSE | 2 | 8825572 | 0.0143582 | -0.0494572 | 0.13982 | 0.723548 |
| rs2208589 | G | A | FALSE | FALSE | 20 | 47408414 | 0.781873 | 0.0363071 | 0.041069 | 0.376669 |
| SNP, single nucleotide polymorphism; eaf, effect allele frequency; se, standard error; pval, p value; F, F-statistic; chr, chromosome; pos, position. | | | | | | | | | | |

| **TABLE S3** List of instrumental variables used in the analyses for the exposure of pre-eclampsia or eclampsia, and corresponding gene-outcome association estimates for all SNPs available in outcome GWAS. | | | | | | | | | | |
| --- | --- | --- | --- | --- | --- | --- | --- | --- | --- | --- |
| **Pre-eclampsia or eclampsia** | | | | | | | | | | |
| SNP | effect_allele | other_allele | eaf | beta | se | pval | R^2^ | F |  |  |
| rs17367504 | G | A | 0.145 | -0.14975 | 0.03066 | 1.03E-06 | 0.000169078 | 23.85520779 |  |  |
| rs11121976 | T | C | 0.162 | -0.14211 | 0.0292 | 1.13E-06 | 0.000167874 | 23.68521977 |  |  |
| rs113653429 | C | T | 0.0336 | 0.27196 | 0.0595 | 4.86E-06 | 0.000148076 | 20.89151698 |  |  |
| rs1226832 | C | G | 0.127 | -0.15446 | 0.03252 | 2.04E-06 | 0.000159894 | 22.55925898 |  |  |
| rs2369286 | A | G | 0.255 | -0.1144 | 0.02474 | 3.75E-06 | 0.000151551 | 21.38191019 |  |  |
| rs10004588 | A | C | 0.0452 | 0.24205 | 0.05247 | 3.97E-06 | 0.000150832 | 21.28050296 |  |  |
| rs10944316 | T | C | 0.0695 | 0.20027 | 0.04298 | 3.17E-06 | 0.000153888 | 21.71164933 |  |  |
| rs60736424 | C | T | 0.284 | -0.1104 | 0.02394 | 4.01E-06 | 0.000150729 | 21.26589657 |  |  |
| rs7388321 | C | G | 0.971 | -0.31406 | 0.06412 | 9.67E-07 | 0.000170034 | 23.99010054 |  |  |
| rs12775642 | A | G | 0.323 | 0.11353 | 0.02313 | 9.22E-07 | 0.000170753 | 24.09150065 |  |  |
| rs4766568 | C | T | 0.16 | -0.14111 | 0.02968 | 2.00E-06 | 0.00016021 | 22.60380923 |  |  |
| rs2912370 | C | T | 0.521 | -0.09989 | 0.02171 | 4.19E-06 | 0.000150048 | 21.16987151 |  |  |
| rs138609024 | C | T | 0.00627 | 0.73836 | 0.15008 | 8.67E-07 | 0.000171549 | 24.20385391 |  |  |
| rs116887748 | T | C | 0.0375 | 0.25939 | 0.05674 | 4.85E-06 | 0.000148127 | 20.89881387 |  |  |
| rs137882343 | T | G | 0.0112 | 0.51261 | 0.10619 | 1.38E-06 | 0.000165161 | 23.30240518 |  |  |
| rs167479 | G | T | 0.574 | 0.10815 | 0.02172 | 6.38E-07 | 0.000175723 | 24.79289542 |  |  |
| rs6060809 | T | C | 0.0348 | 0.26984 | 0.05881 | 4.47E-06 | 0.000149217 | 21.0525225 |  |  |
| rs17572606 | T | C | 0.00985 | 0.54056 | 0.11465 | 2.42E-06 | 0.000157559 | 22.22969666 |  |  |
| **Ovarian cancer** | | | | | | | | | | |
| SNP | effect_allele | other_allele | palindromic | ambiguous | chr | pos | eaf | beta | se | pval |
| rs10004588 | A | C | FALSE | FALSE | 4 | 126390454 | 0.06312 | -0.02246 | 0.02897 | 0.4382 |
| rs10944316 | T | C | FALSE | FALSE | 6 | 88261817 | 0.0428 | 0.006964 | 0.03695 | 0.8505 |
| rs11121976 | T | C | FALSE | FALSE | 1 | 12833428 | 0.1922 | -0.01268 | 0.01726 | 0.4625 |
| rs113653429 | C | T | FALSE | FALSE | 1 | 21557407 | 0.02843 | 0.035 | 0.04166 | 0.4008 |
| rs116887748 | T | C | FALSE | FALSE | 18 | 60358234 | 0.01737 | -0.03587 | 0.06005 | 0.550301 |
| rs1226832 | C | G | TRUE | FALSE | 1 | 45595626 | 0.1289 | 0.01877 | 0.02032 | 0.3556 |
| rs12775642 | A | G | FALSE | FALSE | 10 | 121712667 | 0.3364 | -0.007446 | 0.01491 | 0.6175 |
| rs137882343 | T | G | FALSE | FALSE | 18 | 65010976 | 0.02625 | -0.1169 | 0.05774 | 0.0430002 |
| rs138609024 | C | T | FALSE | FALSE | 17 | 32119136 | 0.01998 | 0.08643 | 0.06432 | 0.179 |
| rs167479 | G | T | FALSE | FALSE | 19 | 11526765 | 0.505 | -0.01297 | 0.01748 | 0.457999 |
| rs17367504 | G | A | FALSE | FALSE | 1 | 11862778 | 0.1662 | 0.0118 | 0.01829 | 0.5187 |
| rs17572606 | T | C | FALSE | FALSE | 22 | 24868172 | 0.02098 | 0.00365 | 0.05482 | 0.9469 |
| rs2369286 | A | G | FALSE | FALSE | 4 | 4595540 | 0.2725 | 0.0008346 | 0.01565 | 0.9575 |
| rs2912370 | C | T | FALSE | FALSE | 15 | 39050654 | 0.5363 | -0.0153 | 0.01486 | 0.3033 |
| rs6060809 | T | C | FALSE | FALSE | 20 | 34717350 | 0.04131 | 0.02959 | 0.03498 | 0.3976 |
| rs60736424 | C | T | FALSE | FALSE | 6 | 154936452 | 0.2395 | 0.03047 | 0.01551 | 0.0494903 |
| rs7388321 | C | G | TRUE | FALSE | 8 | 17939143 | 0.9927 | -0.04923 | 0.08213 | 0.5489 |
| **Cervical cancer** | | | | | | | | | | |
| SNP | effect_allele | other_allele | palindromic | ambiguous | chr | pos | eaf | beta | se | pval |
| rs10004588 | A | C | FALSE | FALSE | 4 | 126390454 | 0.0592086 | -0.121492 | 0.187071 | 0.516053 |
| rs10944316 | T | C | FALSE | FALSE | 6 | 88261817 | 0.0345158 | -0.0458727 | 0.242231 | 0.849799 |
| rs11121976 | T | C | FALSE | FALSE | 1 | 12833428 | 0.172445 | -0.179283 | 0.1166 | 0.124151 |
| rs113653429 | C | T | FALSE | FALSE | 1 | 21557407 | 0.0270477 | 0.285174 | 0.270302 | 0.291417 |
| rs116887748 | T | C | FALSE | FALSE | 18 | 60358234 | 0.0116924 | -0.140766 | 0.416887 | 0.735621 |
| rs1226832 | C | G | TRUE | FALSE | 1 | 45595626 | 0.122092 | 0.0795862 | 0.134874 | 0.555138 |
| rs12775642 | A | G | FALSE | FALSE | 10 | 121712667 | 0.315888 | 0.0347582 | 0.0979264 | 0.722633 |
| rs137882343 | T | G | FALSE | FALSE | 18 | 65010976 | 0.0195004 | -0.0907991 | 0.323121 | 0.778705 |
| rs138609024 | C | T | FALSE | FALSE | 17 | 32119136 | 0.0123932 | 0.434628 | 0.402268 | 0.279944 |
| rs167479 | G | T | FALSE | FALSE | 19 | 11526765 | 0.528339 | 0.0371751 | 0.088344 | 0.673901 |
| rs17367504 | G | A | FALSE | FALSE | 1 | 11862778 | 0.162156 | 0.123774 | 0.119679 | 0.301035 |
| rs17572606 | T | C | FALSE | FALSE | 22 | 24868172 | 0.0141887 | -0.0292691 | 0.376537 | 0.938041 |
| rs2369286 | A | G | FALSE | FALSE | 4 | 4595540 | 0.279852 | 0.0580909 | 0.0989726 | 0.557245 |
| rs2912370 | C | T | FALSE | FALSE | 15 | 39050654 | 0.534468 | 0.0970895 | 0.0911595 | 0.286853 |
| rs4766568 | C | T | FALSE | FALSE | 12 | 111724699 | 0.134693 | 0.012818 | 0.12935 | 0.921063 |
| rs6060809 | T | C | FALSE | FALSE | 20 | 34717350 | 0.0367008 | 0.219065 | 0.234433 | 0.350075 |
| rs60736424 | C | T | FALSE | FALSE | 6 | 154936452 | 0.248366 | -0.00286409 | 0.101961 | 0.97759 |
| rs7388321 | C | G | TRUE | FALSE | 8 | 17939143 | 0.99462157 | -0.454357 | 0.603673 | 0.451658 |
| **Endometrial cancer** | | | | | | | | | | |
| SNP | effect_allele | other_allele | palindromic | ambiguous | chr | pos | eaf | beta | se | pval |
| rs10004588 | A | C | FALSE | FALSE | 4 | 126390454 | 0.0598005 | 0.0298914 | 0.0321652 | 0.35273 |
| rs10944316 | T | C | FALSE | FALSE | 6 | 88261817 | 0.04751625 | 0.0475443 | 0.0415032 | 0.251978 |
| rs11121976 | T | C | FALSE | FALSE | 1 | 12833428 | 0.181234625 | 0.0279149 | 0.0199866 | 0.16251 |
| rs113653429 | C | T | FALSE | FALSE | 1 | 21557407 | 0.028341 | 0.0190657 | 0.0473898 | 0.687451 |
| rs116887748 | T | C | FALSE | FALSE | 18 | 60358234 | 0.01437675 | -0.0985344 | 0.0660884 | 0.135975 |
| rs1226832 | C | G | TRUE | FALSE | 1 | 45595626 | 0.118776875 | -0.0338395 | 0.0237505 | 0.154217 |
| rs12775642 | A | G | FALSE | FALSE | 10 | 121712667 | 0.337389375 | 0.000162199 | 0.0170914 | 0.992428 |
| rs137882343 | T | G | FALSE | FALSE | 18 | 65010976 | 0.0212865 | 0.0266101 | 0.0660386 | 0.686986 |
| rs138609024 | C | T | FALSE | FALSE | 17 | 32119136 | 0.01762875 | 0.00112669 | 0.07116 | 0.987367 |
| rs167479 | G | T | FALSE | FALSE | 19 | 11526765 | 0.520121625 | 0.00762732 | 0.0190058 | 0.688188 |
| rs17367504 | G | A | FALSE | FALSE | 1 | 11862778 | 0.1564785 | 0.00409044 | 0.0209032 | 0.844857 |
| rs17572606 | T | C | FALSE | FALSE | 22 | 24868172 | 0.017822875 | 0.0195114 | 0.0621903 | 0.75372 |
| rs2369286 | A | G | FALSE | FALSE | 4 | 4595540 | 0.281025 | 0.0239153 | 0.0175795 | 0.173701 |
| rs2912370 | C | T | FALSE | FALSE | 15 | 39050654 | 0.53290475 | 0.000663675 | 0.0166949 | 0.96829 |
| rs4766568 | C | T | FALSE | FALSE | 12 | 111724699 | 0.137386375 | 0.0353618 | 0.021995 | 0.107896 |
| rs6060809 | T | C | FALSE | FALSE | 20 | 34717350 | 0.0359875 | -0.0130401 | 0.0409816 | 0.750339 |
| rs60736424 | C | T | FALSE | FALSE | 6 | 154936452 | 0.253394875 | -0.00729891 | 0.017965 | 0.684533 |
| **Uterine fibroids** | | | | | | | | | | |
| SNP | effect_allele | other_allele | palindromic | ambiguous | chr | pos | eaf | beta | se | pval |
| rs10004588 | A | C | FALSE | FALSE | 4 | 126390454 | 0.0592086 | -0.00858566 | 0.0359677 | 0.811335 |
| rs10944316 | T | C | FALSE | FALSE | 6 | 88261817 | 0.0345158 | 0.00456626 | 0.04702 | 0.922637 |
| rs11121976 | T | C | FALSE | FALSE | 1 | 12833428 | 0.172445 | -0.0515152 | 0.0224822 | 0.0219412 |
| rs113653429 | C | T | FALSE | FALSE | 1 | 21557407 | 0.0270477 | -0.00311831 | 0.0521842 | 0.95235 |
| rs116887748 | T | C | FALSE | FALSE | 18 | 60358234 | 0.0116924 | -0.157742 | 0.0790919 | 0.0461067 |
| rs1226832 | C | G | TRUE | FALSE | 1 | 45595626 | 0.122092 | -0.00813337 | 0.0259638 | 0.754084 |
| rs12775642 | A | G | FALSE | FALSE | 10 | 121712667 | 0.315888 | 0.0308306 | 0.0188573 | 0.102061 |
| rs137882343 | T | G | FALSE | FALSE | 18 | 65010976 | 0.0195004 | -0.0486897 | 0.062099 | 0.433002 |
| rs138609024 | C | T | FALSE | FALSE | 17 | 32119136 | 0.0123932 | 0.000324656 | 0.0772534 | 0.996647 |
| rs167479 | G | T | FALSE | FALSE | 19 | 11526765 | 0.528339 | 0.00511119 | 0.0170207 | 0.763954 |
| rs17367504 | G | A | FALSE | FALSE | 1 | 11862778 | 0.162156 | -0.03961 | 0.0230337 | 0.0854944 |
| rs17572606 | T | C | FALSE | FALSE | 22 | 24868172 | 0.0141887 | -0.120249 | 0.0723041 | 0.0962917 |
| rs2369286 | A | G | FALSE | FALSE | 4 | 4595540 | 0.279852 | -0.00162725 | 0.019068 | 0.931992 |
| rs2912370 | C | T | FALSE | FALSE | 15 | 39050654 | 0.534468 | -0.0119011 | 0.0175575 | 0.497874 |
| rs4766568 | C | T | FALSE | FALSE | 12 | 111724699 | 0.134693 | 0.00960469 | 0.0248764 | 0.699425 |
| rs6060809 | T | C | FALSE | FALSE | 20 | 34717350 | 0.0367008 | -0.0211542 | 0.0453222 | 0.640677 |
| rs60736424 | C | T | FALSE | FALSE | 6 | 154936452 | 0.248366 | -0.0192153 | 0.0196521 | 0.328186 |
| rs7388321 | C | G | TRUE | FALSE | 8 | 17939143 | 0.99462157 | 0.204898 | 0.11612 | 0.0776421 |
| **Breast cancer** | | | | | | | | | | |
| SNP | effect_allele | other_allele | palindromic | ambiguous | chr | pos | eaf | beta | se | pval |
| rs10004588 | A | C | FALSE | FALSE | 4 | 126390454 | 0.0588169 | 0.0299978 | 0.0703882 | 0.66998 |
| rs10944316 | T | C | FALSE | FALSE | 6 | 88261817 | 0.0343223 | -0.120632 | 0.092118 | 0.190352 |
| rs11121976 | T | C | FALSE | FALSE | 1 | 12833428 | 0.171907 | -0.0522911 | 0.0439074 | 0.233677 |
| rs113653429 | C | T | FALSE | FALSE | 1 | 21557407 | 0.0271355 | -0.0772912 | 0.102644 | 0.451447 |
| rs116887748 | T | C | FALSE | FALSE | 18 | 60358234 | 0.0119026 | 0.072531 | 0.15428 | 0.638265 |
| rs1226832 | C | G | TRUE | FALSE | 1 | 45595626 | 0.121875 | -0.0498688 | 0.0507534 | 0.325818 |
| rs12775642 | A | G | FALSE | FALSE | 10 | 121712667 | 0.316586 | 0.0168287 | 0.0369161 | 0.648488 |
| rs137882343 | T | G | FALSE | FALSE | 18 | 65010976 | 0.0197073 | -0.101103 | 0.121175 | 0.40408 |
| rs138609024 | C | T | FALSE | FALSE | 17 | 32119136 | 0.0125572 | -0.175165 | 0.151406 | 0.247304 |
| rs167479 | G | T | FALSE | FALSE | 19 | 11526765 | 0.528524 | 0.0171364 | 0.0333076 | 0.60691 |
| rs17367504 | G | A | FALSE | FALSE | 1 | 11862778 | 0.162091 | 0.0618116 | 0.0450113 | 0.169676 |
| rs17572606 | T | C | FALSE | FALSE | 22 | 24868172 | 0.0142952 | -0.0989651 | 0.140679 | 0.481757 |
| rs2369286 | A | G | FALSE | FALSE | 4 | 4595540 | 0.279842 | -0.0238511 | 0.037338 | 0.522959 |
| rs2912370 | C | T | FALSE | FALSE | 15 | 39050654 | 0.534573 | -0.0138318 | 0.0343123 | 0.686863 |
| rs4766568 | C | T | FALSE | FALSE | 12 | 111724699 | 0.134542 | 0.0793939 | 0.0486317 | 0.102563 |
| rs6060809 | T | C | FALSE | FALSE | 20 | 34717350 | 0.0366804 | 0.104876 | 0.0886363 | 0.236721 |
| rs60736424 | C | T | FALSE | FALSE | 6 | 154936452 | 0.248041 | -0.0119351 | 0.038404 | 0.755971 |
| rs7388321 | C | G | TRUE | FALSE | 8 | 17939143 | 0.99459677 | -0.218415 | 0.22662 | 0.335149 |
| SNP, single nucleotide polymorphism; eaf, effect allele frequency; se, standard error; pval, p value; F, F-statistic; chr, chromosome; pos, position. | | | | | | | | | | |

| **TABLE S4** Multivariable Mendelian randomization (MVMR) analysis of gestational hypertension with common gynecologic tumors in European population. | | | | | | |
| --- | --- | --- | --- | --- | --- | --- |
| Outcomes | Methods | Adjustments | nSNP | OR | 95% CI | *p* value |
| Ovarian cancer | Inverse variance weighted | SBP | 350 | 0.947 | 0.885-1.015 | 0.123 |
|  |  | BMI | 408 | 0.964 | 0.910-1.020 | 0.201 |
|  |  | T2DM | 74 | 0.902 | 0.796-1.022 | 0.106 |
|  | MR Egger | SBP | 350 | 0.905 | 0.819-1.000 | 0.049 |
|  |  | BMI | 408 | 0.959 | 0.876-1.049 | 0.356 |
|  |  | T2DM | 74 | 0.924 | 0.826-1.033 | 0.163 |
|  | MVMR median | SBP | 350 | 0.924 | 0.856-0.997 | 0.043 |
|  |  | BMI | 408 | 0.975 | 0.911-1.045 | 0.483 |
|  |  | T2DM | 74 | 0.939 | 0.835-1.056 | 0.296 |
| Cervical cancer | Inverse variance weighted | SBP | 362 | 0.985 | 0.664-1.461 | 0.94 |
|  |  | BMI | 419 | 1.066 | 0.743-1.529 | 0.727 |
|  |  | T2DM | 77 | 1.242 | 0.686-2.250 | 0.474 |
|  | MR Egger | SBP | 362 | 1.192 | 0.668-2.130 | 0.553 |
|  |  | BMI | 419 | 1.169 | 0.666-2.051 | 0.586 |
|  |  | T2DM | 77 | 1.195 | 0.560-2.551 | 0.645 |
|  | MVMR median | SBP | 362 | 0.935 | 0.582-1.503 | 0.782 |
|  |  | BMI | 419 | 1.454 | 0.935-2.259 | 0.097 |
|  |  | T2DM | 77 | 2.423 | 1.066-5.508 | 0.035 |
| Endometrial cancer | Inverse variance weighted | SBP | 367 | 0.983 | 0.907-1.065 | 0.678 |
|  |  | BMI | 426 | 1.007 | 0.942-1.076 | 0.847 |
|  |  | T2DM | 78 | 1.04 | 0.884-1.223 | 0.641 |
|  | MR Egger | SBP | 367 | 0.974 | 0.866-1.096 | 0.665 |
|  |  | BMI | 426 | 0.978 | 0.880-1.087 | 0.681 |
|  |  | T2DM | 78 | 1.059 | 0.857-1.308 | 0.596 |
|  | MVMR median | SBP | 367 | 0.978 | 0.896-1.068 | 0.626 |
|  |  | BMI | 426 | 1.016 | 0.940-1.099 | 0.679 |
|  |  | T2DM | 78 | 1.029 | 0.885-1.197 | 0.706 |
| Uterine fibroids | Inverse variance weighted | SBP | 362 | 0.959 | 0.883-1.041 | 0.315 |
|  |  | BMI | 419 | 0.978 | 0.908-1.054 | 0.564 |
|  |  | T2DM | 77 | 1.018 | 0.886-1.170 | 0.795 |
|  | MR Egger | SBP | 362 | 0.989 | 0.876-1.117 | 0.856 |
|  |  | BMI | 419 | 0.926 | 0.825-1.039 | 0.195 |
|  |  | T2DM | 77 | 0.998 | 0.837-1.191 | 0.984 |
|  | MVMR median | SBP | 362 | 0.953 | 0.868-1.047 | 0.308 |
|  |  | BMI | 419 | 1.006 | 0.923-1.097 | 0.887 |
|  |  | T2DM | 77 | 1.077 | 0.915-1.267 | 0.370 |
| Breast cancer | Inverse variance weighted | SBP | 362 | 1.076 | 0.923-1.253 | 0.35 |
|  |  | BMI | 419 | 1.077 | 0.933-1.242 | 0.313 |
|  |  | T2DM | 77 | 0.989 | 0.794-1.232 | 0.924 |
|  | MR Egger | SBP | 362 | 1.157 | 0.924-1.450 | 0.204 |
|  |  | BMI | 419 | 1.188 | 0.948-1.488 | 0.134 |
|  |  | T2DM | 77 | 1.181 | 0.899-1.550 | 0.233 |
|  | MVMR median | SBP | 362 | 1.048 | 0.875-1.255 | 0.608 |
|  |  | BMI | 419 | 1.09 | 0.924-1.285 | 0.307 |
|  |  | T2DM | 77 | 1.203 | 0.897-1.614 | 0.215 |
| SBP, systolic blood pressure; BMI, body mass index; T2DM, type 2 diabetes mellitus. | | | | | | |

| **TABLE S5** Multivariable Mendelian randomization (MVMR) analysis of pre-eclampsia/eclampsia with common gynecologic tumors in European population. | | | | | | |
| --- | --- | --- | --- | --- | --- | --- |
| Outcomes | Methods | Adjustments | nSNP | OR | 95% CI | *p* value |
| Ovarian cancer | Inverse variance weighted | SBP | 345 | 0.939 | 0.882-1.000 | 0.051 |
|  |  | BMI | 406 | 1.016 | 0.964-1.071 | 0.549 |
|  |  | T2DM | 81 | 0.959 | 0.899-1.023 | 0.202 |
|  | MR Egger | SBP | 345 | 0.956 | 0.872-1.048 | 0.334 |
|  |  | BMI | 406 | 1.019 | 0.944-1.100 | 0.616 |
|  |  | T2DM | 81 | 0.964 | 0.893-1.040 | 0.339 |
|  | MVMR median | SBP | 345 | 0.918 | 0.855-0.985 | 0.018 |
|  |  | BMI | 406 | 0.981 | 0.918-1.049 | 0.561 |
|  |  | T2DM | 81 | 0.925 | 0.835-1.024 | 0.131 |
| Cervical cancer | Inverse variance weighted | SBP | 357 | 0.905 | 0.622-1.316 | 0.601 |
|  |  | BMI | 418 | 0.912 | 0.655-1.270 | 0.588 |
|  |  | T2DM | 85 | 1.467 | 0.960-2.240 | 0.077 |
|  | MR Egger | SBP | 357 | 0.748 | 0.431-1.298 | 0.303 |
|  |  | BMI | 418 | 0.667 | 0.414-1.074 | 0.096 |
|  |  | T2DM | 85 | 1.170 | 0.711-1.925 | 0.536 |
|  | MVMR median | SBP | 357 | 0.910 | 0.577-1.437 | 0.687 |
|  |  | BMI | 418 | 1.053 | 0.686-1.618 | 0.814 |
|  |  | T2DM | 85 | 1.303 | 0.688-2.469 | 0.417 |
| Endometrial cancer | Inverse variance weighted | SBP | 362 | 0.985 | 0.914-1.061 | 0.687 |
|  |  | BMI | 425 | 1.016 | 0.956-1.080 | 0.61 |
|  |  | T2DM | 85 | 1.011 | 0.918-1.113 | 0.827 |
|  | MR Egger | SBP | 362 | 0.963 | 0.863-1.074 | 0.499 |
|  |  | BMI | 425 | 0.979 | 0.897-1.070 | 0.64 |
|  |  | T2DM | 85 | 1.002 | 0.893-1.125 | 0.97 |
|  | MVMR median | SBP | 362 | 0.995 | 0.915-1.083 | 0.905 |
|  |  | BMI | 425 | 1.047 | 0.972-1.128 | 0.233 |
|  |  | T2DM | 85 | 0.997 | 0.883-1.126 | 0.964 |
| Uterine fibroids | Inverse variance weighted | SBP | 357 | 1.055 | 0.976-1.142 | 0.176 |
|  |  | BMI | 418 | 0.993 | 0.927-1.064 | 0.846 |
|  |  | T2DM | 85 | 0.969 | 0.875-1.072 | 0.543 |
|  | MR Egger | SBP | 357 | 1.089 | 0.972-1.220 | 0.144 |
|  |  | BMI | 418 | 0.970 | 0.880-1.070 | 0.556 |
|  |  | T2DM | 85 | 0.998 | 0.886-1.125 | 0.976 |
|  | MVMR median | SBP | 357 | 1.069 | 0.979-1.168 | 0.141 |
|  |  | BMI | 418 | 0.975 | 0.898-1.059 | 0.556 |
|  |  | T2DM | 85 | 0.985 | 0.867-1.119 | 0.818 |
| Breast cancer | Inverse variance weighted | SBP | 357 | 0.956 | 0.827-1.105 | 0.541 |
|  |  | BMI | 418 | 0.932 | 0.818-1.063 | 0.301 |
|  |  | T2DM | 85 | 0.875 | 0.749-1.021 | 0.088 |
|  | MR Egger | SBP | 357 | 0.897 | 0.724-1.110 | 0.318 |
|  |  | BMI | 418 | 0.969 | 0.801-1.171 | 0.739 |
|  |  | T2DM | 85 | 0.894 | 0.744-1.075 | 0.233 |
|  | MVMR median | SBP | 357 | 0.861 | 0.726-1.021 | 0.085 |
|  |  | BMI | 418 | 0.951 | 0.810-1.117 | 0.541 |
|  |  | T2DM | 85 | 0.805 | 0.634-1.022 | 0.075 |
| SBP, systolic blood pressure; BMI, body mass index; T2DM, type 2 diabetes mellitus. | | | | | | |
